# Simultaneous Discovery, Estimation and Prediction Analysis of Complex Traits Using a Bayesian Mixture Model

**DOI:** 10.1371/journal.pgen.1004969

**Published:** 2015-04-07

**Authors:** Gerhard Moser, Sang Hong Lee, Ben J. Hayes, Michael E. Goddard, Naomi R. Wray, Peter M. Visscher

**Affiliations:** 1 Queensland Brain Institute, University of Queensland, Brisbane, Australia; 2 Department of Primary Industries, Biosciences Research Division, Bundoora, Australia; 3 Dairy Futures Cooperative Research Centre, Bundoora, Australia; 4 Faculty of Land and Food Resources, University of Melbourne, Melbourne, Australia; 5 University of Queensland Diamantina Institute, University of Queensland, Translational Research Institute (TRI), Brisbane, Australia; MRC Human Genetics Unit, UNITED KINGDOM

## Abstract

Gene discovery, estimation of heritability captured by SNP arrays, inference on genetic architecture and prediction analyses of complex traits are usually performed using different statistical models and methods, leading to inefficiency and loss of power. Here we use a Bayesian mixture model that simultaneously allows variant discovery, estimation of genetic variance explained by all variants and prediction of unobserved phenotypes in new samples. We apply the method to simulated data of quantitative traits and Welcome Trust Case Control Consortium (WTCCC) data on disease and show that it provides accurate estimates of SNP-based heritability, produces unbiased estimators of risk in new samples, and that it can estimate genetic architecture by partitioning variation across hundreds to thousands of SNPs. We estimated that, depending on the trait, 2,633 to 9,411 SNPs explain all of the SNP-based heritability in the WTCCC diseases. The majority of those SNPs (>96%) had small effects, confirming a substantial polygenic component to common diseases. The proportion of the SNP-based variance explained by large effects (each SNP explaining 1% of the variance) varied markedly between diseases, ranging from almost zero for bipolar disorder to 72% for type 1 diabetes. Prediction analyses demonstrate that for diseases with major loci, such as type 1 diabetes and rheumatoid arthritis, Bayesian methods outperform profile scoring or mixed model approaches.

## Author Summary

Most genome-wide association studies performed to date have focused on testing individual genetic markers for associations with phenotype. Recently, methods that analyse the joint effects of multiple markers on genetic variation have provided further insights into the genetic basis of complex human traits. In addition, there is increasing interest in using genotype data for genetic risk prediction of disease. Often disparate analytical methods are used for each of these tasks. We propose a flexible novel approach that simultaneously performs identification of susceptibility loci, inference on the genetic architecture and provides polygenic risk prediction in the same statistical model. We illustrate the broad applicability of the approach by considering both simulated and real data. In the analysis of seven common diseases we show large differences in the proportion of genetic variation due to loci with different effect sizes and differences in prediction accuracy between complex traits. These findings are important for future studies and the understanding of the complex genetic architecture of common diseases.

## Introduction

Genome wide association studies (GWAS) have been used for three different purposes—to map genetic variants causing variation in a trait, to estimate the genetic variance explained by all the single nucleotide polymorphisms (SNPs) that have been genotyped, and to predict the genetic value or future phenotype of individuals. These analyses are usually performed using different statistical models and methods. To map causal variants usually the SNPs are analyzed one at a time, consequently the failure to account for the effects of other SNPs increases the error variance and thus decreases the power to detect true associations [1,2]. The effects of the SNPs are treated as fixed effects and, to account for the multiple testing, a stringent *p*-value is used, resulting in many false negatives but typically over-estimating the effects of SNPs declared significant [3]. For most traits the significantly associated SNPs only explain a fraction of the heritability, and thus have low predictive power, even when considered in aggregate [4].

To estimate the variance explained by all the SNPs together, all genotyped or imputed SNPs can be included in the model simultaneously with their effects treated as random variables all drawn from a normal distribution with zero mean and constant variance. This gives an unbiased estimate of the variance explained, but all the estimated SNP effects are non-zero [5].

The most accurate method to predict genetic value or phenotype based on the SNP genotypes is to fit all SNPs simultaneously treating the SNP effects as drawn from a prior distribution that matches the true distribution of SNP effects as closely as possible [4,6]. We do not know the true distribution of effect sizes but a mixture of normal distributions can approximate a wide variety of distributions by varying the mixing proportions [7]. Erbe et al. [8] used this prior and included one component of the mixture with zero variance. A similar model was proposed by Zhou et al. [9] but with a mixture of two normal distributions, one with a small variance and one with a larger variance.

The models used for prediction can also be used to map variants associated with phenotype and to estimate the total variance explained by the SNPs. Because they fit all SNPs simultaneously and account for LD between SNPs, they should have greater power to detect associations, find less false negatives and give unbiased estimates of the larger SNP effects. They can also provide information about the genetic architecture of the trait from the hyper-parameters of the distribution of SNP effects.

Here we use a Bayesian mixture model (called BayesR [8]) to dissect genetic variation for disease in human populations and to construct more powerful risk predictors. We show how this method can shed light on the genetic architecture underlying complex diseases as well as demonstrating its ability to map SNPs associated with disease and estimate the genetic variance explained by the SNPs collectively. The approach was evaluated on simulated and real data of seven case-control traits from the Welcome Trust Case Control Consortium. We assessed the power to correctly identify causal and associated variants, to estimate SNP-based heritability and the accuracy to predict future outcomes. Results from BayesR are compared with a traditional single-SNP GWAS analysis, a linear mixed-effects modeling approach [5,10–12] and a Bayesian sparse linear mixed model [9].

## Results

### Hierarchical Bayesian Mixture Model (BayesR)

In most GWAS studies the number of markers is very large and notably *p*>>*n*. This requires some kind of variable selection, either by discarding unimportant predictors or by shrinking their effects to zero. We used a Bayesian mixture model and *a priori* assumed a mixture of four zero mean normal distributions of SNP effects (*β*), where the relative variance for each mixture component is fixed [8]:

$$\begin{array}{l} p\left(\beta_{j}|\pi ,\sigma_{g}^{2}\right)=\pi_{1}\times N\left(0,0\times \sigma_{g}^{2}\right)+\pi_{2}\times N\left(0,10^{-4}\times \sigma_{g}^{2}\right)+\\ \pi_{3}\times N\left(0,10^{-3}\times \sigma_{g}^{2}\right)+\pi_{4}\times N\left(0,10^{-2}\times \sigma_{g}^{2}\right). \end{array}$$

Here, **π** are the mixture proportions which are constrained to sum to unity and **$\sigma_{g}^{2}$** is the additive genetic variance explained by SNPs. Sparseness is included into the model by setting the effect and variance of the first mixture component to zero. Instead of fixing $\sigma_{g}^{2}$ at a pre-specified value [8], we estimate a hyper-parameter for the genetic variance from the data. We compare BayesR with traditional single-SNP GWAS analyses [13], a linear mixed-effects modeling approach (LMM) [5,10–12] and a Bayesian sparse linear mixed model (BSLM) [9,14].

### Results from Simulated Data using Real Genotypes

We used real genotype data of 287,854 SNPs measured on 3,924 individuals to simulate quantitative phenotypes with heritabilities equal to 0.2, 0.5, and 0.8. Causal effects were drawn from three groups of effect sizes, the first containing 10 SNPs with moderate effects, the second containing 310 SNPs with smaller effect, and a large group of 2,680 SNPs representing a polygenic component (S1 Fig.), where the definitions of moderate, small and polygenic effect size match those of the prior assumptions of BayesR. Note that the contribution of each mixture to heritability is not known *a priori* (S2 Fig.).

#### Identifying associated SNPs

**Fig 1:**
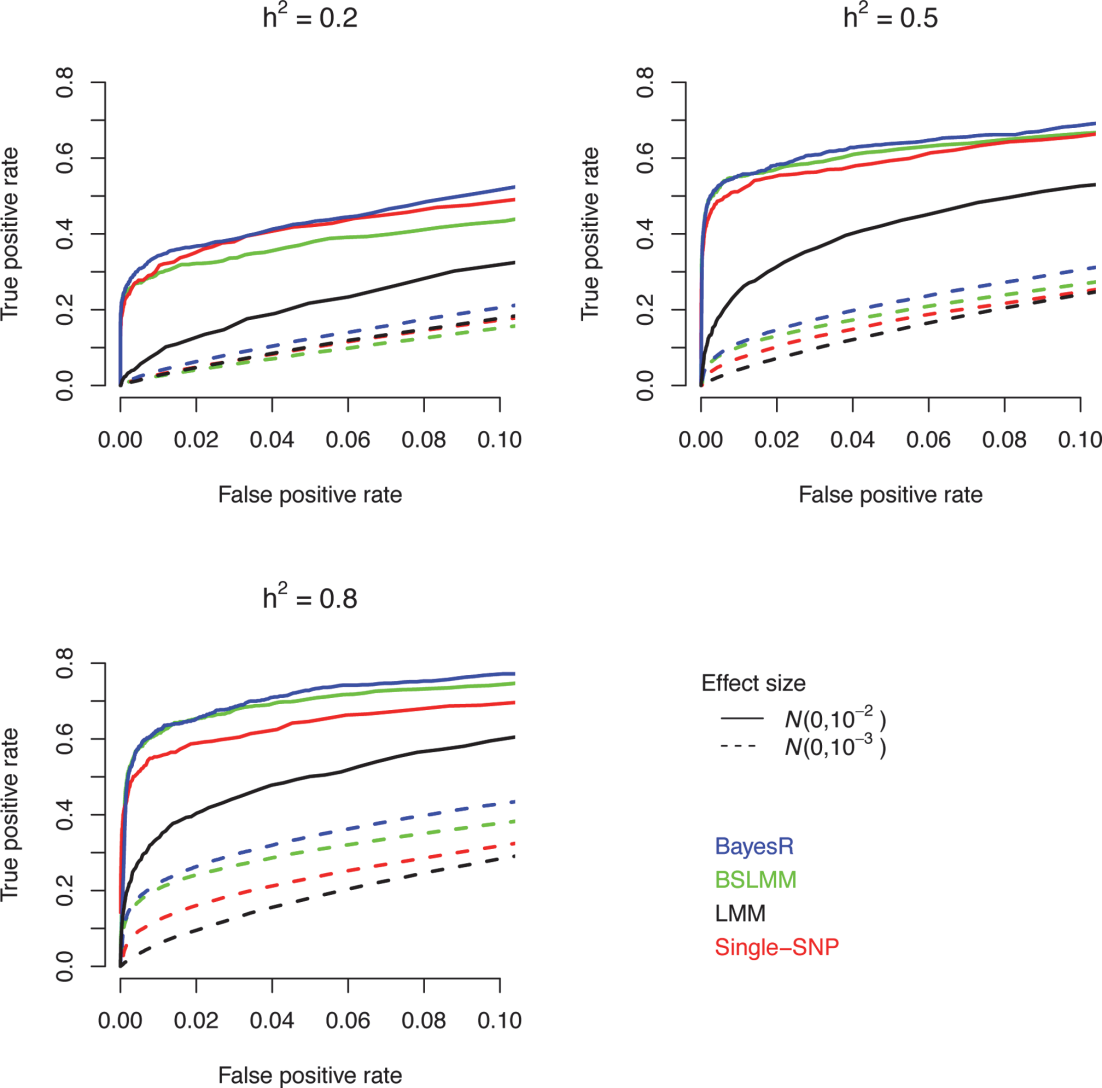
Comparison of causal variant identification accuracy of BayesR, BSLM, LMM and single-SNP analysis in simulated data. Shown is the true positive rate as a function of false positive rate for correct identification of regions (250kb) containing causative SNPs. Simulations are based on real SNP data of 3,924 individuals genotyped for 287,854 SNPs. The total number of causative SNPs was 3,000 with 10 (solid line), 310 (dotted line) and 2,680 effects sampled from a zero mean normal distribution with variance 10^−2^, 10^−3^, and 10^−4^, respectively. Trait heritabilities (*h* ^*2*^) were 0.2, 0.5 and 0.8.

Comparisons between methods are assessed on their ability to identify genomic regions of 250kb containing causal SNPs. This was done because the multi-SNP methods tend to split a QTL effect, even if large, across SNPs in LD with the QTL. Moreover, it may be improper to declare a non-causative SNP in LD with the causal variant a false positive. However, we loosely use the term causal variant for convenience. Fig. 1 shows that only the segments harboring the largest SNPs were accurately identified at a meaningful false positive rate. The ability to accurately locate causal variants decreased with decreasing effect size of the SNP. The power to map accurately the 2,680 polygenic SNPs was very low. BayesR yielded more true positive regions than the other methods across the three heritabilities. Qualitatively similar results were obtained using shorter and longer genome regions (S3 Fig.). Both Bayesian approaches outperformed single-SNP analysis at higher heritability (*h* ^*2*^ = 0.5 and 0.8). A likely explanation for the gain of the multi-SNP Bayesian methods is an increase in power to detect subsequent causal SNPs after the strongest associations have been accounted for.

#### SNP-based heritability

**Fig 2:**
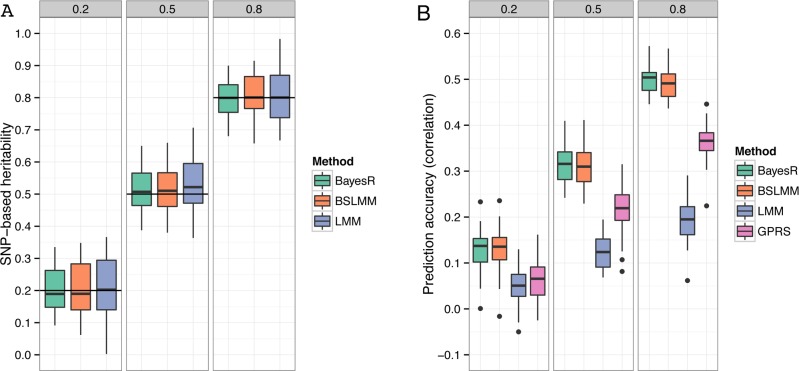
Comparison of performance of BayesR, BSLMM, LMM and GPRS in simulated data. (A) Distribution of SNP-based heritability estimates. The horizontal lines indicate the true heritability. GPRS does not provide estimates of heritability. (B) Distribution of the correlation coefficient between true and predicted phenotype. Simulations are based on real SNP data of 3,924 individuals genotyped for 287,854 SNPs. The total number of causative SNPs was 3,000 with 10, 310 and 2,680 effects sampled from a zero mean normal distribution with variance 10^−2^, 10^−3^, and 10^−4^, respectively. Trait heritabilities (*h* ^*2*^) were 0.2, 0.5 and 0.8. The single boxplots display the variation in estimates among 50 replicates.

In general all methods gave unbiased estimates of the true heritability values. The mean estimates (± standard deviation) of the proportion of variance explained by typed SNPs ($h_{g}^{2}$) for heritabilities of 0.2, 0.5 and 0.8 were 0.20 (±0.065), 0.52 (±0.067) and 0.80 (±0.055) for BayesR (Fig. 2A). Estimates of BSLMM and LMM were 14 and 32% less accurate (larger standard deviations) than BayesR. This may reflect the fact that the assumed effect size distributions of BayesR closely matched those of the true model. The analyzed simulated SNP sets included the causal variants; hence $h_{g}^{2}$ equals *h* ^2^. The true SNP-based heritability would be unknown when the causal SNPs were excluded from the panel due to incomplete LD between makers and causative variants.

#### Prediction accuracy

Each data set of the simulation was randomly split into a training sample containing 80% of individuals and a validation sample containing the remaining 20%. Prediction accuracy was measured with Pearson’s correlation coefficient between observed and predicted phenotype in the validation sample. The mean (± standard deviation) correlation coefficient for BayesR was 0.13 (±0.041), 0.32 (±0.038) and 0.50 (±0.032) for simulated *h* ^*2*^ of 0.2, 0.5 and 0.8, respectively (Fig. 2B). BayesR and BSLMM yielded almost the same accuracies and their advantage over LMM and GPRS was relatively large. For all heritabilities LMM generated the lowest accuracies.

#### Genetic architecture

**Fig 3:**
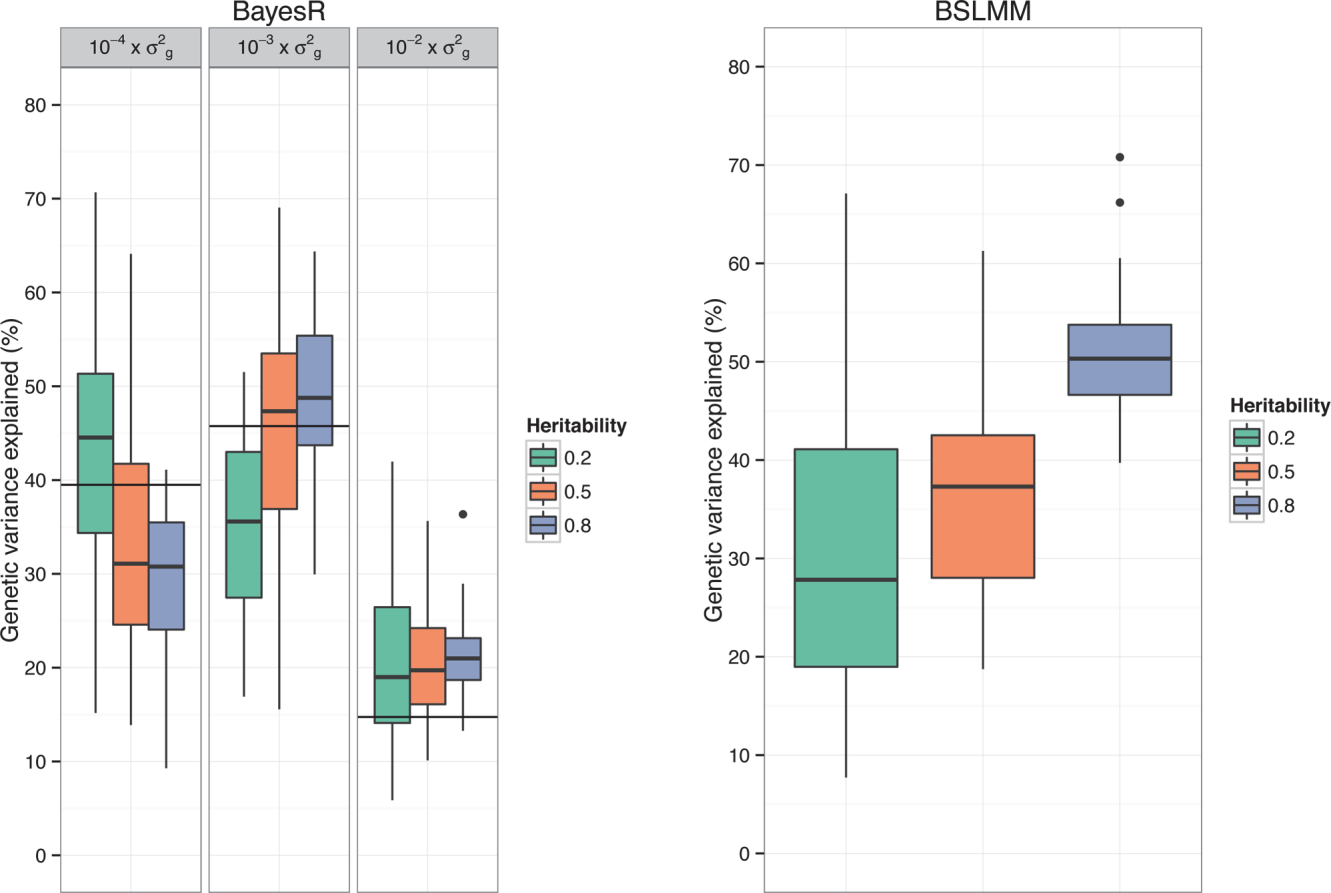
Genetic architecture inferred using BayesR and BSLMM in simulated data. Shown is the proportion of total genetic variance explained by each mixture component for BayesR and the relative contribution of SNPs with an effect above the polygenic component for BSLMM. Simulations are based on real SNP data of 3,924 individuals genotyped for 287,854 SNPs. The total number of causative SNPs was 3,000 with 10, 310, and 2,680 effects sampled from a zero mean normal distribution with variance 10^−2^, 10^−3^, and 10^−4^, respectively. The horizontal lines indicate the expected contribution by the 10 (right), 310 (middle) and 2,680 (left) SNPs to the genetic variance. Trait heritabilities (*h* ^*2*^) were 0.2, 0.5 and 0.8. The single boxplots display the variation in estimates among 50 replicates.

A feature of BayesR is that it can be used to quantify how many SNPs affect a trait and their contribution to the total genetic variance (Fig. 3). We calculated the variance in each mixture component as the sum of the square of the sampled effect sizes of SNPs allocated to each component. Mean (± standard deviation) contribution to genetic variance of components with SNP variance $10^{-4}\times \sigma_{g}^{2},10^{-3}\times \sigma_{g}^{2}$ and $10^{-2}\times \sigma_{g}^{2}$ was 43% (±14.0), 36% (±9.8) and 21% (±8.3) for *h* ^*2*^ = 0.2, 34% (±12.6), 45% (±11.9) and 21% (±6.7) for *h* ^*2*^ = 0.5 and 30% (±8.0), 49% (±7.6) and 21% (±4.0) for *h* ^*2*^ = 0.8. Note that the true underlying mixture is not identifiable (S2 Fig.), however the proportion of variance explained by each mixture component showed good correspondence to the simulated genetic architecture. Estimates were generally not very precise, which is partly due to the large sampling variance when simulating SNP effects. BSLMM provides an estimate of the relative contribution of SNPs with an effect above the polygenic component, and this estimate showed a strong increase with increasing heritability of the trait (Fig. 3).

### Sensitivity Analyses

In additional simulations, under models that ranged from very sparse to polygenic and using alternate parametric models for the effect-size distribution, we assessed how our prior assumption may affect parameters estimates and interpretation of results (S2 Text). To cover a wide range of architectures from very sparse to polygenic, we sampled 10, 100, 1,000, 10,000, and 20,000 causal SNPs either from a standard normal distribution or a gamma distribution with shape 0.44 and scale 1.66 [15,16]. In general estimates of heritability from all methods were robust across the wider range of settings (S1 Table). Heritability estimates of LMM were unbiased, even under scenarios where its modeling assumptions were not met. BayesR and BSLMM showed a small upward bias under very sparse scenarios and BayesR slightly underestimated heritability under highly polygenic models. BayesR estimates had the smallest variance in the very sparse setting (10 causative variants) despite prior specifications that did not closely correspond to the true model.

Similar to the previous results using real genotype data, where the prior model closely matched the analysis model of BayesR, prediction accuracies from BayesR and BSLMM were highest and both methods performed almost the same across all the scenarios (S2 Table). LMM was the least accurate method with the exception of scenarios including 10,000 and 20,000 SNPs. BayesR and BSLMM outperformed GPRS, with the exception of the scenarios involving 10 causative SNPs. These results show that the mixture models are more powerful than GPRS, even in the case of LE markers where the single SNP method might be expected to do very well.

Inferences of BayesR about the genetic architecture were consistent with the underlying model and provided insights into the genetic architecture (S4–S5 Figs.). Posterior inference of the BayesR model for the scenario including 10 causative SNPs, which is poorly supported by the BayesR prior, provided strong evidence to revise the prior model. As for the 287K data, BayesR and BSLMM outperformed LMM and GRPS in finding causal variants in all scenarios (S6 Fig.).

### Analyses of WTCCC Data

In addition to simulated data we assessed the performance of BayesR for seven diseases of the Welcome trust case control consortium (WTCCC [17]). These data were previously used to estimate heritability [18,19] and for risk prediction [14,20–22].

#### SNP-based heritability

**Fig 4:**
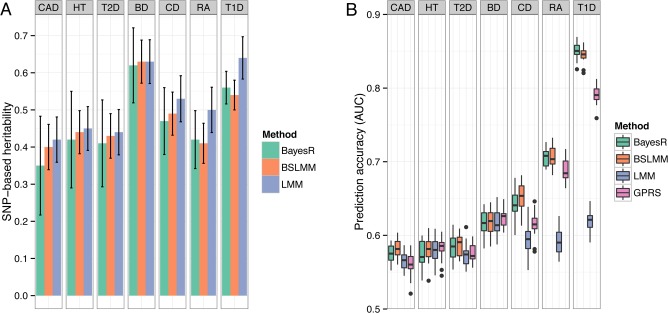
Comparison of performance of BayesR, BSLMM, LMM and GPRS in WTCCC data. (A) Estimates of SNP-based heritability on the observed scale. Antennas are standard deviations of posterior samples for BayesR and BSLMM or standard errors for LMM. GPRS does not provide estimates of heritability. (B) Distribution of the area under the curve (AUC). The single boxplots display the variation in estimates among 20 replicates. In each replicate, the data set was randomly split into a training sample containing 80% of individuals and a validation sample containing the remaining 20%.

We report $h_{g}^{2}$ for the diseases in WTCCC on the liability scale (S3 Table), but make comparisons on the observed scale since the controls are common between traits so that comparisons reflect the underlying genetic architecture in the cases samples. For five of the seven traits (BD, CAD, CD, HT, RA), estimates of $h_{g}^{2}$ were very similar between methods with estimates from BayesR slightly lower than BSLMM and LMM (Fig. 4A). For RA and T1D, which have large associations with alleles in the major histocompatibility complex, $h_{g}^{2}$ from the Bayesian methods was much smaller compared to LMM. Estimates of BayesR were less consistent (indicated by larger posterior standard deviations), particularly for traits with a large polygenic contribution to variance, such as BD and HT.

#### Accuracy and bias of prediction

We created 20 random 80/20 splits for each disease and assessed accuracy by computing the area under the curve (AUC [23]). The predictive performance for all seven diseases is shown in Fig. 4B. Mean (± standard deviation) of AUC scores of BayesR were 0.58 (±0.012) for CAD, 0.58 (±0.017) for HT, 0.58 (±0.017) for T2D, 0.62 (±0.017) for BD, 0.64 (±0.018) for CD, 0.71 (±0.012) for RA and 0.85 (±0.011) for T1D. Although BayesR performed well for some diseases, prediction performance assessed in case/control data suffers from ascertainment bias [24], because the prevalence in the general populations is much lower than the prevalence in the case/control study, where cases are substantially overrepresented. We therefore also report prediction performance of the methods while accounting for prevalence (S4 Table). BayesR and BSLMM performed equally well across the seven traits with a mean AUC of 0.56 and outperformed GPRS and LMM in diseases where the original study identified relatively strong associations (CD, RA, T1D) [17]. GPRS and LMM had comparable prediction accuracy for traits where the known risk loci have effects of small individual magnitude (HT, BD). Prediction accuracy of LMM increased with increasing heritability, but there was no direct relationship between estimates of $h_{g}^{2}$ and predictive performance for the other methods.

The regressions of phenotype on predicted value for GPRS were considerably larger than one (Table 1), showing that the difference in the predictions of a pair of individuals is smaller than the difference in their phenotypes. Predictions from the other methods showed little or no bias. An unbiased predictor is necessary when genomic predictions are to be combined with different information sources (*e*.*g*. sex, smoking status, BMI etc.) for risk prediction.

**Table 1:** Regression of phenotype on predicted value for BayesR, BSLMM, LMM and GPRS in WTCCC data.

Disease	BayesR		BSLMM		LMM		GPRS	
BD	1.13	(0.280)	1.09	(0.236)	1.12	(0.246)	2541	(1403.5)
CAD	0.96	(0.216)	0.92	(0.179)	0.99	(0.187)	1529	(919.0)
CD	0.98	(0.137	1.01	(0.134)	1.05	(0.341)	35.4	(15.50)
HT	1.08	(0.461)	0.98	(0.313)	1.04	(0.356)	2124	(1041.9)
RA	0.99	(0.080)	0.98	(0.096)	1.07	(0.330)	33.1	(20.2)
T1D	1.00	(0.037)	0.99	(0.096)	1.03	(0.169)	57.3	(14.8)
T2D	0.92	(0.265)	0.94	(0.188)	0.99	(0.322)	1894	(789.9)

For prediction assessment we performed 20 random 80/20 splits for each trait. The values parentheses are standard deviations over 20 replicates.

#### Genetic architecture

A feature of BayesR is that it estimates the number of associated SNPs along with their variance explained. The posterior mean of the number of SNP fitted in WTCCC varied considerably between traits (S5 Table). The number of SNPs was comparatively low for T1D, where 2,633 individual SNPs explained the total genetic variance. The largest number of SNPs was included in the model for BD (N = 9,411) of which more than 99% had very small effects (effect size$10^{-4}\times \sigma_{g}^{2}$).

**Fig 5:**
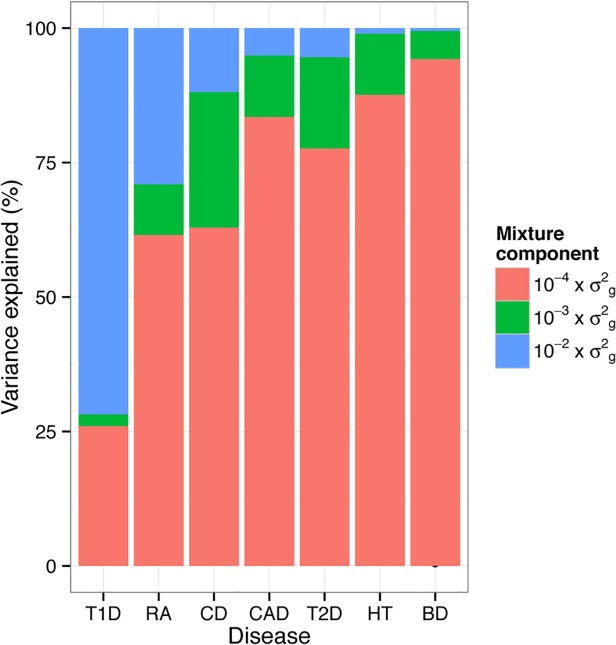
Genetic architecture underlying seven traits in WTCCC inferred using BayesR. Proportion of additive genetic variation contributed by SNPs with different effect sizes. The colored bars partition the genetic variance in contributions from each mixture class. The proportion of variance in each mixture component was calculated as the sum of the square of the sampled effect sizes of the SNPs allocated to each component divided by the sum of the total variance explained by SNPs.

The proportion of variance explained by each mixture component varied markedly across the seven diseases (Fig. 5). Large numbers of SNPs with small effects ($10^{-4}\times \sigma_{g}^{2}$) contributed the majority of the genetic variance explained for BD (94.3%), HT (87.6%), CAD (83.5%) and T2D (77.6%). A substantial proportion of the total variance was explained by a small number of SNPs with larger effect sizes ($10^{-2}\times \sigma_{g}^{2}$) for T1D (71.8%), RA (29.0%) and CD (11.9%). As might be expected prediction accuracy of BayesR was also the highest for these traits and credible intervals indicate that genetic trait architecture is inferred with reasonable precision for most traits (S7 Fig.).

**Fig 6:**
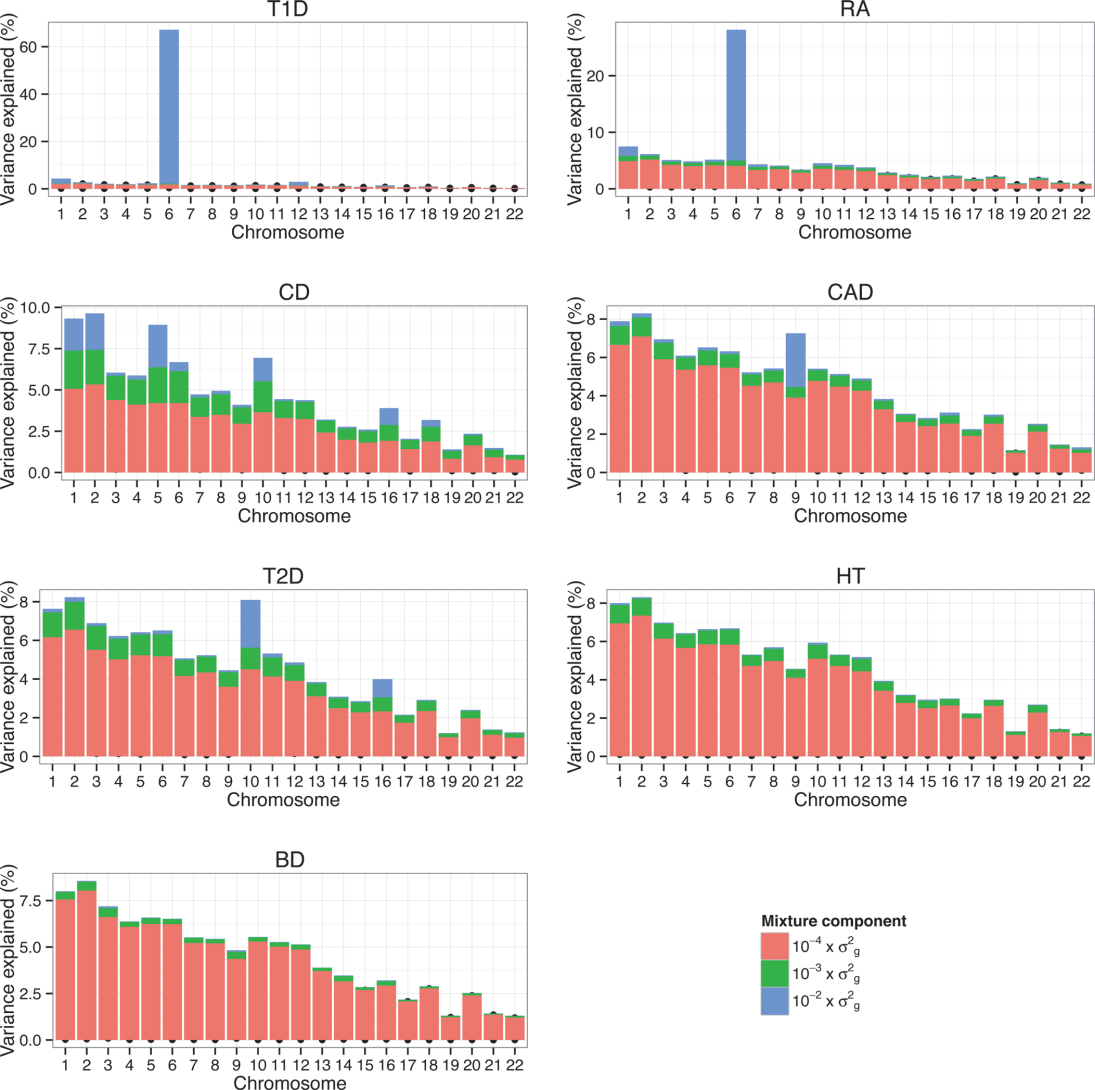
Proportion of genetic variance on each chromosome explained by SNPs with different effect sizes underlying seven traits in WTCCC. Proportion of additive genetic variation contributed by individual chromosomes and the proportion of variance on each chromosome explained by SNPs with different effect sizes. For each chromosome we calculated the proportion of variance in each mixture component as the sum of the square of the sampled effect sizes of the SNPs allocated to each component divided by the sum of the total variance explained by SNPs. The colored bars partition the genetic variance in contributions from each mixture class.

We also assessed the proportion of additive genetic variation contributed by individual chromosomes and the proportion of variance on each chromosome explained by SNPs with different effect sizes (Fig. 6). Estimates of the variance explained by each chromosome were largely related to the length of the chromosome with the majority of variation consistent with a polygenic architecture. Differences in the contribution of single chromosomes on individual traits were mostly due to SNPs with large effect ($10^{-2}\times \sigma_{g}^{2}$) and to a lesser extent to SNPs with smaller effects ($10^{-3}\times \sigma_{g}^{2}$). On the whole, regions and chromosomes that explained large proportions of the SNP-based variance coincide well with the regions that showed the strongest association signals in the original study (Table 3 and Fig. 4 in WTCCC study published in [17]). One example is chromosome 9 that harbors SNPs with large effect on CAD and the most significant SNP (rs1333049) was located within a 44kb region spanned by 6 SNP with a posterior inclusion probability of 1.2 (sum of the posterior inclusion probabilities of the 6 SNP). The region accounted for 27.2% of the genetic variance of chromosome 9. We estimated that chromosome 6 contributed 67.2% of the genetic variance in T1D, which is larger than the 47–58% reported using LMM [11,18]. More than 96% of the variance explained by chromosome 6 in T1D was due to SNPs with large effects. Chromosome 6 accounted for 28.1% of the genetic variance in RA which is slightly less than ∼33% using LMM [18].

#### Computational demand

Computing time is important, particularly with the tremendous number of markers in many human SNP data sets. The average running time required for each method to perform prediction analysis for BD and T1D (chosen as examples because of their different genetic architectures) is shown in Table 2. Computation time depends on a number of factors, including programming language and software environment, and for the sampling based methods in particular on the number of iterations used. We did not investigate in detail how many iterations are sufficient and ran BSLMM with its default value of 1,000,000 sampling steps and BayesR for 50,000 iterations. We observed only minor differences in the posterior distributions between replicated chains and interpreted this as evidence that the algorithm converged (S8–S9 Figs.). The requirements of Bayes-R were several orders of magnitude higher than BSLMM when compared on a per-iteration basis; nevertheless, the run time of BayesR was competitive for the data sets considered here. LMM was computationally less demanding than the other methods. LD-based clumping accounted for most of the computational burden of GRPS and had to be repeated 10 times in our cross-validation scheme. Computing time for BayesR scales linearly with the number of SNPs and to reduce the computational burden of BayesR we changed the per-iteration MCMC scheme as follows. For the first 5,000 cycles the effect of each SNP was sampled per iteration. After this we did not sample all SNPs in each MCMC iteration, but updated SNP effects (in random order) only until we had sampled 500 SNPs with non-zero effects. SNPs not updated in the current iteration kept their effect sizes from the previous MCMC cycle. The idea behind this is based on two observations. Firstly, SNPs with larger effects appear more quickly in the model. Secondly, most of the calculation time is spent on sampling small SNP effects in and out of the model to mimic the ‘polygenic ‘ component, but which individual SNP is retained in the model has minimal effect on the posterior. We found that the ‘500 SNPs’ strategy generated similar results to the “All SNPs’ strategy (S6 Table), but that the computational burden was significantly reduced by a factor of 3 to 6 (Table 2).

**Table 2:** Mean computation time (in hours) of various methods for risk prediction in WTCCC.

	BayesR		BSLMM	LMM	GPRS
Trait	All SNPs	500 SNPs			
T1D	17.76 (0.19)	5.94 (1.19)	8.59 (0.63)	0.24 (0.06)	256.5 (10.07)
BD	18.40 (0.17)	3.33 (0.65)	10.27 (3.66)	0.25 (0.02)	202.9 (36.52)

Values are means with standard deviations in parentheses. Computations were performed using a computing cluster with Intel E5-2670 processors with 2.66 GHz CPUs. Shown is the time taken to analyze 10 replicates of the 80/20 split data for BD and T1D. BayesR (All SNPs) samples each SNP effect in each MCMC iteration. BayesR (500 SNPs) uses a modified sampling scheme.

## Discussion

We have presented a single model for analysis of GWAS that maps associated variants, estimates the genetic variance explained by the SNPs collectively, describes the genetic architecture of the trait and predicts phenotype from SNP genotypes. The framework we present applies a Bayesian hierarchical model to human complex traits based on the assumption of a prior distribution that SNP effects come from a mixture of more than two normal distributions. The procedure clusters markers in groups with distinct genetic values where each SNP explains 0.01, 0.1, or 1% of $\sigma_{g}^{2}$ and a group of SNPs with zero effect. Instead of fixing the variance component $\sigma_{g}^{2}$ to a pre-specified value as in Erbe et al. [8] we treat $\sigma_{g}^{2}$ as unknown and estimate it from the data. This is because the shrinkage of SNP effects is affected by $\sigma_{g}^{2}$ and determining the amount of shrinkage a priori can have negative impact on performance [9,16].

BayesR showed good performance in estimating the SNP-based heritability across a wider range of simulated genetic architectures (Fig. 2A, S1 Table) and estimates were similar to BSLMM and LMM for diseases of the WTCCC study (Fig. 4A). If the primary interest is to estimate SNP based heritability, LMM is faster and approximately unbiased under different disease architectures [9,11,18]. BayesR can provide more accurate estimates under certain architectures, for example when effect sizes follow skewed distributions, which is the case for many human diseases[4].

For phenotype prediction BayesR was as accurate as BSLMM which outperformed various other approaches in the study of Zhou et al. [9]. Qualitatively, the main difference between the methods considered here is that the BayesR model is sparse, which seems intuitively appealing, as not every genotyped SNP is likely to be in LD with causative variants. For example, often in GWAS the primarily focus is not on estimating the relative contribution of each genetic variant, but whether or not a particular variant contributes at all. Sparseness and good performance make BayesR an attractive alternative to currently available methods.

The Bayesian framework incorporates model uncertainty by averaging over many different competing models [25], and this allows for more robust inferences about the genetic architecture. The posterior inclusion probability can be directly interpreted as the probability that a variant is an risk factor with a certain effect size [26], which is more intuitive to interpret than an association of zero or one based on a *p*-value from single-SNP analysis. Our simulations showed that SNPs with high inclusion probabilities have a high probability of being a causal or associated variant (Figs. 1, S3, S6) and an increase in performance of BayesR to identify smaller SNPs that are currently difficult to detect in single SNP GWAS [1,27,28].

Predictions of phenotypes from BayesR, BSLMM and LMM were unbiased (Table 1). Unbiasedness of a disease predictor is important for practical implementations [29,30], yet often ignored when developing GPRS derived from GWAS summary statistics.

We applied BayesR directly to the WTCCC data treating the binary outcome coded 0/1 as the response in an ordinary linear regression. The predicted phenotypes can then be taken as the probability of being a case and heritability estimates can be transformed to liability of disease scale [11]. The model can be extended to binary or ordered categorical traits by fitting a liability model [31], but improvements are expect to be negligible [27,32].

By quantifying the contribution of SNPs and their effect sizes, BayesR can be used to make inferences about the underlying genetic architecture of complex phenotypes (Figs. 3, 5, 6, S4). In our analysis of WTCCC, we found that most of the SNPs had a zero effect (>96%), inconsistent with the ‘infinitesimal model’ [33], but that thousands each explain a small proportion of the total genetic variance and these estimates suggest a substantial contribution of a polygenic component to these common diseases. However inferences did vary between diseases, with fewer loci contributing to the genetic variance for T1D and RA than for the other traits. This difference is mainly a result of large effects associated with variants in the MHC for T1D and RA. Furthermore the variance explained by larger SNPs (effect size$10^{-2}\times \sigma_{g}^{2}$) varied markedly between chromosomes and between diseases, ranging from 73% of $h_{g}^{2}$ for T1D to 0.6% for BD. Consistent with other studies the variance explained by individual chromosomes was largely related to its length [34–36], although chromosomes of similar length showed large variability across diseases, which was due to SNPs with larger effects.

We caution against over-interpretation of our results as they relate to genetic architecture [37]. Inevitably the specified mixture model that effect sizes come from four mixture distributions is very simplistic. Nonetheless, since the WTCCC diseases all utilize the same control samples, the differences between diseases allows comparative interpretation and the genetic architectures agree well with the findings in the original [17] and subsequent studies [11,18,22]. However, in practice the true effect size distribution is unknown. We used the same mixture distribution as prior as Erbe et al. [8], where it showed good mixing between SNPs, but alternative prior distributions may lead to better performance. Priors may be influential, however, simulating a large number of different genetic architectures, we found that in general results were not very sensitive to our modeling assumptions and that inferences of BayesR about the genetic architecture were consistent with the underlying simulated genetic architecture (S4 Fig.). Using a distribution with variance $10^{-4}\times \sigma_{\text{g}}^{2}$ seemed a reasonable choice for the effect size of ‘polygenic’ SNPs (S5 Fig.). How much of the heavier tail of the distribution can be distinguished from zero effects depends on sample size. For much larger data sets adding more classes, for example one with variance$10^{-5}\times \sigma_{\text{g}}^{2}$, might help to interpret the data.

In addition to the caveats relating to the specific mixture model we emphasize that the inference drawn on SNP effects and genetic architecture is from observed SNPs and not on the causal variants directly. The true but unknown pattern of correlation between unobserved causal variants and genotyped SNPs will impact the inference about genetic architecture. Nevertheless, the comparison across the seven diseases, for which the genotyped SNPs are the same, demonstrates large differences in SNPs effects, variance explained and prediction accuracy, reflecting real differences in the distribution of effect sizes at causal variants.

Incorporating markers beyond the small number of risk variants identified at genome wide significance has the potential to increase the predictive performance of risk models [4,38]. Our results on predicting disease risk in WTCCC are consistent with recent analysis [9,20–22] that demonstrated that predictive ability of polygenic models is trait specific, depending on heritability and genetic architecture. Furthermore, our results extend beyond previous reports of the impact of genetic architecture on genetic risk prediction, most of which have relied on hypothetical effect-size distributions or used results from risk predictions to inform about genetic architecture [38,39]. Here we infer genetic architecture directly from entire GWAS data, which can contribute to our understanding of complex disease and our ability to assess the power of future GWAS depending on the underlying disease architecture. We observed that the pattern of SNP-based heritability did not follow the same pattern as those of AUC. In particular, heritability was not a good indicator of prediction performance for BayesR and BSLMM. For traits where common SNP account for a large proportion of the SNP based heritability (T1D, RA, CD), predictive accuracy was much higher for the two Bayesian methods compared to LMM and GPRS.

BayesR has proved feasible in the WTCCC data set with ∼300,000 markers, but much larger data sets are currently being collected. Computing time increases linearly with the number of SNPs, however, runtime for large SNP sets can be reduced by avoiding redundant computations through filtering of SNPs that are in perfect or high linkage disequilibrium with at least another SNP. The savings can be quite substantial, ranging from 9–22% (*r* ^*2*^ = 1) to 34–58% (*r* ^*2*^>0.80) for the Hapmap3 panel [40], depending on the ancestry of the population [41]. Computing performance can further be improved by running multiple MCMC chains instead of a single long chain. Moreover, computing time of the ‘500 SNPs’ implementation does not increase linearly with the number of SNP after the first 5,000 cycles, thus reducing computational burden even more for larger data sets. However, less arbitrary approaches should be developed.

For very large datasets Bayesian-like estimation using MCMC might be infeasible altogether, and fast alternative Bayesian estimation procedures are required [42,43]. On the other hand, the use of a simple Gibbs sampling scheme provides great flexibility in effects size distributions by selecting the number and the variances of the mixture. We illustrated the flexibility of the method by partitioning the genetic variance into contribution of SNPs with different effect sizes by chromosomes. This model can easily be extended to allow for different prior probabilities of the mixture distribution for each chromosome [44], to include dominant genetic variation [28], to partitioned variance attributable to SNPs by annotation [34], or to include prior biological knowledge in genomic analysis and prediction [45].

We found little difference between BayesR and BSLMM in prediction performance, however, differences seem likely when individual effects sizes can be estimated more accurately with increase in sample size. For instance, as sample size increases and genome sequence data is analyzed, causal variants explaining only 0.1% of genetic variance will be identified. An advantage of BayesR is that most SNPs have near zero effect and so could be deleted from prediction of future phenotype in practice. Improvements can also be expected when the prior induced mixture distribution more closely captures the actual distribution of effect sizes. It has been shown in simulation studies [46] that models that include all genetic variants do not take full advantage of high-density marker data if the number of causal SNPs is small, while approaches with an implicit feature selection do.

In conclusion, we proposed and applied a flexible Bayesian mixture model that simultaneously estimates effect size of all SNPs, the genetic variation captured by SNPs and maximizes prediction accuracy. We demonstrate the ability of such a model to dissect genetic architecture and partition genetic variation. The method is highly flexible, can be applied to sequence data and can incorporate prior biological knowledge.

## Materials and Methods

### Statistical Framework

Phenotypes are related to markers with a standard linear regression model

$$y=1_{n}\mu +X\beta +\varepsilon ,$$

where **y** is a *n*-dimensional vector of phenotypes, **1**_n_ is a *n*-dimensional vector of ones, *μ* is the general mean, **X** is an *n*×*p* matrix of genotypes encoded as 0, 1 or 2 copies of a reference allele. The vector **β** is a *p*-dimensional vector of SNP effects and *ε* is a *n*-dimensional vector of residuals, $\varepsilon ∼N\left(0,I\sigma_{e}^{2}\right)$ with **I** being a *n*×*n* identity matrix.

### BayesR

The BayesR model assumes that the SNP effects come from a finite mixture of *K* components so that the probability of the *β* effects conditional on the variance of the components $\sigma^{2}=\left(\sigma_{1}^{2},\ldots ,\sigma_{K}^{2}\right)$ and the mixture proportions **π** = (*π*_1_, …, *π*_*K*_) which are constrained to be positive and to sum to unity:

$$p\left(\beta |\pi ,\sigma^{2}\right)=\overset{K}{\underset{k=1}{\sum }}\pi_{k}N\left(\beta |0,\sigma_{k}^{2}\right),$$

where $N\left(\beta |0,\sigma_{k}^{2}\right)$ denotes the density function of the univariate normal distribution with mean 0 and variance$\sigma_{k}^{2}$. The Bayesian approach requires the assignment of prior distributions to all unknowns in the model. We followed Erbe et al. [8] and *a priori* assumed a mixture of four zero mean normal distributions, where the relative variance for each mixture component is fixed:

$$\begin{array}{l} p\left(\beta_{j}|\pi ,\sigma_{g}^{2}\right)=\pi_{1}\times N\left(0,0\times \sigma_{g}^{2}\right)+\pi_{2}\times N\left(0,10^{-4}\times \sigma_{g}^{2}\right)+\\ \pi_{3}\times N\left(0,10^{-3}\times \sigma_{g}^{2}\right)+\pi_{4}\times N\left(0,10^{-2}\times \sigma_{g}^{2}\right). \end{array}$$

Here, $\sigma_{g}^{2}$ is the additive genetic variance explained by SNPs. Sparseness is included into the model by setting the effect and variance of the first mixture component to zero. A key difference in our implementation of BayesR from previous application [8] is that we estimate a hyper-parameter for $\sigma_{g}^{2}$ from the data, rather than fixing the marker variance at a pre-specified value. MCMC estimation of the unknown parameters $\left(\mu ,\pi ,\beta ,\sigma_{g}^{2},\sigma_{e}^{2}\right)$ used a Gibbs scheme to sample values from each unknown parameter’s conditional posterior distribution. Details of the sampling procedure are outlined in S1 Text.

### Simulated Data

Simulations were used to assess the accuracy of estimates of model parameters and of inferences provided by BayesR. The first study represents a typical genome-wide association study and uses real genotype data to capture the correlation between SNPs. Moreover, in GWAS most SNPs are not in LD with causative variants and effect size distribution of causative variants is skewed toward smaller effects.

Here we used genotype data of 3,924 Australian individuals [5]. After quality control, imputation of missing genotypes at each loci and removal of SNPs with a minor allele frequency less than 1%, 287,854 measured SNPs remained. The effects sizes of causal SNPs were assumed to come from a series of three zero mean normal distributions with the number of SNPs in each class falling in inverse proportion to the size of the effect. First we randomly selected 3,000 SNPs to be causal. Large effect sizes were drawn for 10 SNPs by sampling from a normal distribution with variance *σ* ^*2*^ = 10^−2^, moderate effect sizes were generated for 310 SNPs by sampling from a *N*(0,10^−3^) distribution and the effects of the remaining 2,680 SNP were generated from a *N*(0, 10^−4^) distribution. Residual effects for each individual (*e*_*i*_) were obtained by sampling from a normal distribution with mean 0 and with variance chosen to accomplish heritabilities of 0.2, 0.5 or 0.8. The simulated phenotype for a single individual was then obtained as follows:

$$y_{i}=\overset{10}{\underset{j=1}{\sum }}w_{ij}\times \beta_{j}+\overset{310}{\underset{j=11}{\sum }}w_{ij}\times \beta_{j}+\overset{2968}{\underset{j=311}{\sum }}w_{ij}\times \beta_{j}+e_{i},$$

where $w_{ij}=\left(x_{ij}-2p_{j}\right)/\sqrt{2p_{j}\left(1-p_{j}\right)}$ the centered and scaled genotype and *x*_*ij*_ is the number of copies of the reference allele (0, 1, 2) at SNP *j* for individual *i* with *p*_*j*_ being the frequency of the reference allele in the sample. Sampling from this mixture distribution resulted in a fat-tailed distribution of effect sizes (S1 Fig.), where large, moderate and small effects contributed around 14%, 46% and 40% of the total genetic variance. Fifty replicates were analysed for each of the three heritabilities and a different set of 3,000 SNPs was selected for each replicate. In each replicate the sampled 3,000 SNP effects were randomly assigned to the selected markers. Note that the contribution of each causal SNP to heritability depends on its frequency, so that the true number of SNPs in each mixture component of the BayesR model and the contribution of each mixture to heritability are not known *a priori* (S2 Fig.).

### Sensitivity Analysis

In the simulation using real genotype data, phenotypes were generated under a model that very closely matched the prior specifications for BayesR. To investigate how the prior assumption may affect parameters estimates and interpretation of results we performed additional simulations, including scenarios where we created mismatches between modeling assumptions and simulated genetic architectures. To avoid the problem of differentiating between causal variants and non-causal SNPs in LD with causal variants we simulated 20,000 independent SNPs in a sample of 5,000 individuals. Genotypes of SNP *j* were generated by sampling from a binomial distribution with *n* = 2 (number of successes) and success probability *p*_*i*_, where *p*_*i*_ was sampled from a univariate distribution with interval [0.05, 0.5]. We simulated 10, 100, 1,000, 10,000, and 20,000 causal SNPs to cover a wide range of architectures from very sparse to polygenic. Effect sizes were sampled either from a standard normal distribution or a gamma distribution with shape 0.44 and scale 1.66 as in [15,16] and residual effects were added to achieve a heritability of 0.5. Sampling from a gamma distribution generates fewer large and more small effects than the standard normal [16].

### WTCCC Data

We analyzed 7 traits of the Welcome Trust Case Control Consortium (WTCCC) study [17]. Following previous analyses of the data [11,18] we performed strict QC on SNP data using PLINK [13]. First, we removed individuals with > 2% missing genotypes. For each of the 7 case and the two control data sets we removed loci with frequency of the minor allele < 0.01 and SNP with missingness > 1%. After combining each case and the two control sets into 7 trait case/control studies, SNPs significant at 5% for differential missingness between cases and controls and SNP significant at 5% for Hardy-Weinberg equilibrium were removed. Relatedness testing was performed using a pruned set of SNPs with LD of *r* ^2^ <0.05, pairs of subjects with estimated relatedness > 0.05 were identified and one member of each relative pair was removed at random. Principal components of the genomic relationship matrix were estimated with the same set of pruned SNP using the software GCTA [10] and all phenotypes analyzed were the residuals of case-control status following linear regression on the first 20 principal components. After QC the data included 1,851 cases of bipolar disorder (BD), 1,906 cases of coronary artery disease (CAD), 1,731 cases of Crohn’s disease (CD), 1,905 cases of hypertension (HT), 1,837 cases of rheumatoid arthritis (RA), 1,953 cases of type 1 diabetes (T1D), 1,902 cases of type 2 diabetes (T2D), and 2,910 to 2,918 controls depending on the trait. The number of genotypes ranged from 296,718 for BD to 305,967 for CD.

### Other Methods

#### Single-SNP GWAS analysis

Single SNP-trait association analyses were performed using a linear regression model in PLINK [13]. A commonly used method to build prediction models from single-SNP GWAS analyses is genomic risk profiling [38], where SNP effect sizes estimated in one population are used to build a multi-SNP prediction model to generate a genomic profile risk score (GPRS) for each individual in another population. Applying GPRS requires the choice of an appropriate *p*-value threshold used for selecting SNPs into the predictor. We used 10-fold cross-validation to derive the optimal *p*-value threshold for each replicate of the data used for prediction analyses. First, the training data (80% of the total sample for each replicate) was divided in K = 10 non-overlapping folds of equal size. Then GWAS was performed using K-1 folds of data and later SNPs were pruned for independent associations using the “clump” procedure in PLINK, with a pairwise linkage disequilibrium cutoff of *r* ^*2*^<0.25 within a 500kb window. Based on various *p*-value thresholds (0.001, 0.005, 0.01, 0.05, 0.10, 0.15,…, 1.0) an increasing number of SNPs were selected in the predictor. At each value of the threshold the accuracy of predicting the phenotypes in the left-out fold was recorded. This process was repeated *K* times so that every fold was left out once. The *p*-value threshold that yielded the highest average accuracy of prediction in the *K* test sets was then used for the prediction model after estimating SNP effects from the full training set.

#### Linear mixed model (LMM)

We used the software GCTA [10] for linear mixed model analysis. LMM assumes that all SNP effects are drawn from the same normal distribution. In GCTA this is implemented by an equivalent model in which a genomic relationship matrix estimated from the SNPs describes the covariance between the genetic values of individuals [5]. The method is often referred to as GBLUP (genomic best linear unbiased prediction) when used to estimate breeding values of related individuals from marker data in plant and animal breeding, assuming that variances are known without error. However, it is less commonly used for prediction of unrelated individuals in humans [12]. We will refer to the method as LMM as in Zhou et al. [9], but note that its main motivation in human applications is estimation of individual SNP effects and not prediction of aggregate genomic values of individuals. For prediction we estimated genetic values directly fitting the covariance between the genetic values of training and validation individuals. For the mapping of causal variants we used the—blup-snp option in GCTA to transform the BLUP solutions for individuals into the BLUP solutions for SNPs.

#### Bayesian sparse linear mixed model (BSLMM)

BSLMM [9] is a hybrid of the classical polygenic model and sparse regression models. It assumes that effects come from a mixture of two normal distributions, with each genetic variant having at least a small effect on phenotype (polygenic component) and only a fraction of these having an additional effect (sparse component). We fit BSLMM using the GEMMA software [14].

### Identifying Causal SNPs

We compared the ability of BayesR, BSLMM and LMM and single-SNP to identify causal variants. For the simulated 287K data we focused on the SNPs with large and moderate effect sizes sampled from *N*(0,10^−2^) and *N*(0,10^−3^), respectively. Although, small effects together contributed ∼40% to genetic variance, power to identify ‘polygenes’ with our sample size was expected to be effectively zero. Similar to Guan and Stephens [27] we computed a measure of evidence of association between a genome segment and phenotype. This was done because the multi-SNP methods have the tendency to dilute a QTL effect across SNPs in LD with the QTL. For single-SNP analyses we used the minimum of the *p*-values of the SNPs within a region as evidence of association. The sum of the absolute effect sizes of SNPs within a region was used for LMM. The GEMMA software that implements BSLMM outputs the posterior probability of a SNP to have an effect above the polygenic background and we summed these probabilities over the SNPs within a segment. BayesR provides separate inclusion probabilities for an individual SNP to fall in each mixture component. We used the sum of the posterior inclusion probabilities that SNPs are allocated to effect size classes $10^{-2}\times \sigma_{\text{g}}^{2}$ and $10^{-3}\times \sigma_{\text{g}}^{2}$ as evidence measure. In BayesR the polygenic component is ‘mimicked’ by SNPs assigned to the mixture class with small effects size ($10^{-4}\times \sigma_{\text{g}}^{2}$) and was therefore not included in the calculation. We divided the genome in non-overlapping segments of ∼ 250kb size. For each method we selected a series of cutoff values for the evidence measure and considered all segments containing a causative variant that exceed the cutoff value as true positives and all other regions exceeding the cutoff value as false positives. We then plot the true positive rate against the false positive rate averaged over two different starting positions for the first window (0, 125kb). In the simulations using uncorrelated SNPs we assessed the methods on their ability to identify individual SNPs rather than regions. We used similar measures of evidence of association, except for BayesR where we used the posterior probability of the SNP being included in the model (i.e. 1- posterior inclusion probability of class$0\times \sigma_{\text{g}}^{2}$).

### Accuracy and Bias of Prediction

We assessed predictive performance in the simulated data and the WTCCC data. In the simulated data, each replicate was randomly split into a training sample containing 80% of individuals and a validation sample containing the remaining 20%. For the WTCCC data we generated 20 random 80/20 splits for each trait. We use Pearson’s product moment correlation statistic as measure of predictive ability in the simulated data. The accuracy of risk prediction in WTCCC was assessed by the area under the curve (AUC) [23]. We also report the slope of the regression of phenotypes on the predictions. A slope different from one indicates bias in the prediction. A slope of unity from a regression of phenotype on predictor implies that the predictor is calibrated correctly on the scale of absolute risk, which matters in genomic medicine applications, in particular when the genetic predictor is combined with non-genetic factors (*e*.*g*. gender, smoking status, BMI etc.) for risk prediction.

### Implementation

For BayesR, BSLMM and LMM we centered and scaled each column of the genotype matrix to have mean zero and unit variance in all analyses. The data was analyzed using our BayesR software implemented in Fortran. The software is available at http://www.cnsgenomics.com/software/. Prior assumptions for BayesR were as described above (see also S1 Text). For all analyses a chain length of 50,000 was used with the first 20,000 samples as burn-in. Posterior estimates of parameters are based on 3,000 samples drawing every 10^th^ sample after burn-in. GEMMA was run with its default setting of 1,000,000 sampling steps using the first 100,000 as burn-in. The only default parameter we changed was lowering the minor allele frequency threshold to 0.001, to ensure that no SNP was deleted from the model when 80% of the data was used for training.

### Web Resources

The URLs for data presented herein are as follows:

BayesR, http://www.cnsgenomics.com/software/

GCTA, http://www.cnsgenomics.com/software/

GEMMA, http://home.uchicago.edu/xz7/software.html

PLINK, http://pngu.mgh.harvard.edu/∼purcell/plink/

## Supporting Information

S1 Text Detailed description of BayesR. (DOCX)

S2 Text Sensitivity analysis. (DOCX)

S1 Fig Probability density function of the mixture distribution (red) used to sample SNP effects and probability density function of a normal distribution with the same mean and variance (black). (PDF)

S2 Fig Contribution of causal SNPs to heritability in the simulation scenario using real genotypes. SNPs are ranked according to their contribution to heritability calculated as 2*p*(1 – *p*)*β* ^2^, where *p* is the allele frequency and *β* the effect of the SNP. The total number of causative SNPs was 3, 000 with 10, 310 and 2,680 SNP effects sampled from a zero mean normal distribution with variance 10^−2^, 10^−3^, and 10^−4^, respectively. Trait heritabilities (*h* ^*2*^) were 0.2, 0.5 and 0.8.
(PDF)

S3 Fig Comparison of causal variant identification accuracy of BayesR, BSLM, LMM and single-SNP analysis in simulated data using 100kb and 500kb regions. Shown is the true positive rate as a function of false positive rate for correct identification of 100kb (A) and 500kb (B) regions containing causative SNPs. Simulations are based on real SNP data of 3,924 individuals genotyped for 287,854 SNPs. The total number of causative SNPs was 3,000 with 10 (solid line), 310 (dotted line) and 2,680 effects sampled from a zero mean normal distribution with variance 10^−2^, 10^−3^, and 10^−4^, respectively. Trait heritabilities (*h* ^*2*^) were 0.2, 0.5 and 0.8.
(PDF)

S4 Fig Proportion of total genetic variance explained by each mixture component for different simulated genetic architectures. The proportion of variance in each mixture component was calculated as the sum of the square of the sampled effect sizes of the SNPs allocated to each component divided by the sum of the total variance explained by SNPs. Genotype data was simulated for 20,000 uncorrelated SNPs and 5,000 individuals. Genetic architectures ranging from very sparse to highly polygenic were generated by sampling 10, 100, 1,000, 10,000, or 20,0000 SNP effects either from a standard normal distribution or a gamma distribution with shape 0.44 and scale 1.66. Trait heritability was 0.5. Results are based on 50 replicates for each scenario.
(PDF)

S5 Fig Posterior inclusion probabilities (PIP) of SNPs for different simulated genetic architectures. SNPs are ranked according to their contribution to heritability. Genotype data was simulated for 20,000 uncorrelated SNPs and 5,000 individuals. Genetic architectures ranging from very sparse to highly polygenic were generated by sampling 10, 100, 1,000, 10,000, or 20,0000 SNP effects either from (A) a standard normal distribution or (B) a gamma distribution with shape 0.44 and scale 1.66. Trait heritability was 0.5. Results are based on 50 replicates for each case.
(PDF)

S6 Fig Comparison of causal variant identification accuracy of BayesR, BSLM, LMM and single-SNP analysis for different simulated genetic architectures. Shown is the true positive rate as a function of false positive rate to identify causative SNPs. Genotype data was simulated for 20,000 uncorrelated SNPs and 5,000 individuals. 10, 100, 1,000, or 10,000 SNP effects were sampled either from a standard normal distribution (solid line) or a gamma distribution with shape 0.44 and scale 1.66 (dashed line). Trait heritability was 0.5. Results are based on 50 replicates for each scenario.
(PDF)

S7 Fig Proportion of the SNP-based variance explained by each mixture component of BayesR for seven traits in WTCCC. The variance in each mixture component was calculated as the sum of the square of the sampled effect sizes of SNPs allocated to each component. Vertical lines denote 95% credible intervals.
(PDF)

S8 FigComparison of posterior density of the SNP-based heritability (${\text{h}}_{\text{g}}^{2}$) of four independent MCMC chains for BD and T1D in WTCCC.The MCMC chain was run for 50,000 cycles with the first 20,000 samples discarded as burn-in. Posterior estimates of parameters are based on 3,000 samples drawing every 10th sample after burn-in. None of the four runs indicated apparent convergence problems.(PDF)

S9 Fig Trace-plots of the posterior number of SNP in each mixture component of four independent MCMC chains for BD and T1D in WTCCC. The MCMC chain was run for 50,000 cycles with the first 20,000 samples discarded as burn-in. Posterior estimates of parameters are based on 3,000 samples drawing every 10th sample after burn-in. None of the four runs indicated apparent mixing problems.
(PDF)

S1 Table Comparison of estimated heritability from BayesR, BSLMM and LMM for different simulated genetic architectures. (PDF)

S2 Table Comparison of prediction accuracy of BayesR, BSLMM, LMM and GPRS for different simulated genetic architectures. (PDF)

S3 Table Comparison of SNP-based heritability estimates of BayesR, BSLMM and LMM in WTCCC data on the liability scale. (PDF)

S4 Table Percentage of total variance on the liability scale explained in WTCCC data, corrected for ascertainment. (PDF)

S5 Table Estimates of model size and number of SNPs in each mixture component by BayesR for seven traits in WTCCC. (PDF)

S6 Table Posterior means from BayesR using the full (All SNPs) and a reduced MCMC scheme (500 SNPs). (PDF)
