# Supplementary material for: Simultaneous Discovery, Estimation and Prediction Analysis of Complex Traits Using a Bayesian Mixture Model
Source: PLoS Genet. 2015 Apr 7;11(4):e1004969. doi: 10.1371/journal.pgen.1004969 (PMC4388571; doi:10.1371/journal.pgen.1004969)
Supplement: S1 Text — (DOCX) [file pgen.1004969.s001.docx]

**Text S1 Detailed description of BayesR**

***Priors***

The Bayesian approach requires the assignment of prior distributions to all unknowns in the model. The population mean *μ*, was assigned an uninformative uniform prior density. We used the same four-distribution mixture of the SNP effects as Erbe et al. [[1](#_ENREF_1)]

The mixing proportions $\mathbf{\pi}= \left(\pi_{1}, \ldots, \pi_{K}\right)$ are given a symmetric Dirichlet prior i.e. $p\left(\pi_{1}, \ldots, \pi_{4}\right) \sim D\left(\delta, \ldots, \delta\right),$ with *δ*=1. Note that Erbe et al. [[1](#_ENREF_1)] assigned a known value to the variance of all SNP effects $(\sigma_{g}^{2})$, whereas here $\sigma_{g}^{2}$ is informed from the data. The prior for $\sigma_{g}^{2}$is chosen to be of a scaled inverse $-\chi^{2}$distribution, Inv$-\chi^{2}\left(v_{0},S_{0}^{2}\right)$ with known hyperparameters $v_{0}$ and$S_{0}^{2}$. A scaled inverse $-\chi^{2}$distribution was also assumed for $\sigma_{e}^{2}.$ We used flat priors for the hyperparameters for both variances (*v*_o_ = -2 and $S_{0}^{2}$= 0).

***Gibbs sampling***

On the basis of the prior specification, Gibbs sampling was used to generate samples from the posterior distributions of the parameters (using |. to denote conditioned on the data and all other parameters). The Gibbs sampler proceeds as follows:

1. Sample the overall mean from the full conditional posterior distribution

$$\mu|. \sim N\left(n^{-1}\sum_{i=1}^{n} \left(y_{i}- \sum_{j=1}^{p} x_{ij}\beta_{j}\right),\frac{\sigma_{e}^{2}}{n}\right)$$

2. Calculate the probability that SNP *j* is in distribution *k.* The likelihood of SNP *j* being in component *k* is:

$$LogL\left(j,k\right)=\log\left(\pi_{k}\right)- \frac{1}{2\sigma_{e}^{2}} \left(\sum_{i=1}^{n} \tilde{y}_{i}^{2}- \mu_{j,k}\sum_{i=1}^{n} \left(x_{ij}\tilde{y}_{i}\right)^{2} \right)-\frac{1}{2}\text{logV},$$

where $\tilde{y}_{i}$ is the phenotype of individual *i* corrected for the overall mean and the effects of all markers in the model, except marker *j*.

$$\tilde{y}_{i}= \left(y_{i}- \mu-\sum_{l\neq j}^{p} x_{il}\beta_{l}\right)$$

and

$$\mu_{jk}=\frac{\sum_{i=1}^{n} x_{ij} \tilde{y}_{i}}{\sum_{i=1}^{n} x_{ij}^{2}+\sigma_{e}^{2}/\sigma_{k}^{2}}$$

logV is the likelihood of the reduced model including only the effect of SNP *j* and an residual effect:

$\text{logV}=\left|n \text{log}\left(\sigma_{e}^{2}\right)+log\left(\frac{\sigma_{k}^{2}}{\sigma_{e}^{2}}\sum_{i=1}^{n} x_{ij}^{2}+1\right)\right|.$Then the probability of SNP *j* being in distribution *k* is

$$\Pr\left(x_{j\in k}\right)=1/\sum_{l=1}^{K} exp\left[L\left(i,l\right)-L\left(i,k\right)\right].$$

Based on a value sampled from a uninform distribution assign component *k* to SNP *j*.

3. Sample the regression coefficient for SNP *j* from mixture component *k* from the full conditional posterior distribution.

$$\beta_{jk}|. \sim N\left(\mu_{jk}, S_{k}^{2}\right) ,$$

where

$$S_{k}^{2}=\frac{\sigma_{e}^{2}}{\sum_{i=1}^{n} x_{ij}^{2}+\sigma_{e}^{2}/\sigma_{k}^{2}}$$

and $\mu_{jk}$ as above.

4. Repeat step 2 and 3 for SNP *j+*1,..,*p*.

5. Sample $\sigma_{g}^{2}$ from the full conditional posterior distribution:

$$\sigma_{g}^{2}|. \sim\text{Inv}-\chi^{2}\left(v_{0}+m_{g},\frac{m_{g}\sum_{j=1}^{p} \beta_{j}^{2} + v_{o}S_{0}^{2}}{v_{0}+m_{g}}\right),$$

where *m_g_* is the number of SNPs included in the current model.

6. Sample $\sigma_{e}^{2}$ from the full conditional posterior distribution:

$$\sigma_{e}^{2}|. \sim\text{Inv}-\chi^{2}\left(v_{0}+n,\frac{\sum_{i=1}^{n} \left(y_{i}- \mu-\sum_{j=1}^{p} x_{ij}\beta_{j}\right)^{2}+v_{o}S_{0}^{2}}{v_{o}+n}\right)$$

7. Update the mixing proportion by sampling from the posterior:

$$\pi|.\sim\text{ Dirichlet}\left(m_{1}+\delta,\text{ }m_{2}+\delta,m_{3}+\delta,m_{4}+\delta\right),$$

where *m*_1_*,…m*_4_ are the number of markers in each distribution.

7. Compute new $\sigma_{k}^{2}$ of the mixture components

$$\sigma_{k}^{2}\sim\left\{\begin{matrix} \pi_{1} \times0\times\sigma_{g}^{2} \\ \pi_{2}\times10^{-4}\times\sigma_{g}^{2} \\ \pi_{3 }\times10^{-3}\times\sigma_{g}^{2} \\ \pi_{4} \times10^{-2}\times\sigma_{g}^{2} \end{matrix}\right..$$

8. Randomly permute the order of SNPs to provide global moves and to increase mixing.

***Computational efficiency gain***

Updating maker effects requires the computations of $\tilde{y}_{i}$ at step 2 of the algorithm. For example, for sampling the *j*^th^ marker effect, it is considerably more efficient to compute $\tilde{y}_{i}= \left(y_{i}- \mu-\sum_{l\neq j}^{p} x_{il}\beta_{l}\right)$ in the form of $\tilde{y}_{i}=\varepsilon_{i}+x_{ij}\beta_{j}$. If the $\varepsilon_{i}$ are stored then $x_{ij}\beta_{j}$ can be added to the residual for use in sampling the new marker effect $\beta_{j}$, and once this is done the new residual is available by subtraction of the updated $x_{ij}\beta_{j}$ from $\tilde{y}_{i}$ (*e.g*., $\varepsilon_{i}=\tilde{y}_{i}-x_{ij}\beta_{j}).$

***MCMC implementation***

For all analyses the Markov chain was run for 50,000 cycles with the first 20,000 samples discarded as burn-in. Posterior estimates of parameters are based on 3,000 samples drawing every 10^th^ sample after burn in.

***Posterior analysis from the MCMC output***

After having generated samples from the posterior distributions model parameters are estimated by the sample means of their posterior probabilities respectively. Estimates of $\mu, \mathbf{\pi}, \beta,\sigma_{g}^{2},\sigma_{e}^{2}$ are averages over different regression models drawn conditional on different model vectors, known as “Bayesian model averaging”. The posterior inclusion probability (PIP), defined as the proportion of iterations that included a specific marker in the model, was used as a measure of the ability of the model to identify associated SNPs.

***Predictions***

Based on SNP effect estimates obtained from the training population phenotypes of the validation sample were predicted as:

$$\hat{y}_{i}=\mu+\sum_{j\in\beta_{j}>0} w_{ij}\hat{\beta}_{j}$$

where $\hat{\beta}_{j}$ is the estimated effect of SNP *j*, $w_{ij}=\left(x_{ij}-2p_{j}\right)/\sqrt{2p_{j}\left(1-p_{j}\right)}$ and *x_ij_* is the number of copies of the reference allele (0,1,2) at SNP *j* for individual *i* with *p_j_* being the frequency of the reference allele in the training population.

1. Erbe M, Hayes BJ, Matukumalli LK, Goswami S, Bowman PJ, et al. (2012) Improving accuracy of genomic predictions within and between dairy cattle breeds with imputed high-density single nucleotide polymorphism panels. J Dairy Sci 95: 4114-4129.
