# Supplementary material for: Simultaneous Discovery, Estimation and Prediction Analysis of Complex Traits Using a Bayesian Mixture Model
Source: PLoS Genet. 2015 Apr 7;11(4):e1004969. doi: 10.1371/journal.pgen.1004969 (PMC4388571; doi:10.1371/journal.pgen.1004969)
Supplement: S2 Text — (DOCX) [file pgen.1004969.s002.docx]

**Text S2 Sensitivity analysis**

For both normally and gamma distributed effects there was good correspondence between the inferred values and the true heritability of 0.5 for all methods (Table S1). Figure S4 shows that inferences from BayesR about the genetic architecture were consistent with the underlying model and provided insights into the genetic architecture. For example, when sampling 1,000 SNPs from *N*(0,1) most of the genetic variance will be explained by SNPs allocated to the component with variance 1/1000=10^-3^. The plots of posterior inclusion probabilities of individual SNPs against their association with phenotype (Figure S5) indicated that the fitted distributions performed well in separating the SNPs into groups. The plots also demonstrate that for highly polygenic models there was considerable uncertainty if an individual SNP should be included in the model or not.

BayesR performed well for architectures with 10 and 20,000 causative SNPs, both settings that are outside the model space for the non-null effects covered by the prior. For the 10 SNPs models we observed that on average very few SNPs (16.1 and 10.5 for standard normal and gamma distribution, resp.) were assigned to the largest mixture component. Multiplying the number of SNPs with the genetic variance assigned to each marker (i.e. 10^-2^) gives values of ~16.1% and 10.5% for the expected proportion of variance explained by SNPs with variance . The expected proportions are much smaller than the actual posterior estimates (Figure S4) and provide strong evidence that the prior assumptions are wrong and to revise the modeling assumptions.

Table S2 compares the prediction accuracy of the four methods. Prediction accuracy was measured as correlation between true and predicted phenotypes. Prediction performance was almost identical between BayesR and BSLMM. LMM performed poorly in all situations except for scenarios including 10,000 and 20,000 SNPs. BayesR and BSLMM outperformed GPRS, with the exception of the scenarios involving 10 causative SNPs.

Similar to the simulation using real genotypes, BayesR and BSLMM performed as well or better than LMM and GRPS in correctly identifying causal variants (Figure S6).
