# Supplementary material for: Simultaneous Discovery, Estimation and Prediction Analysis of Complex Traits Using a Bayesian Mixture Model
Source: PLoS Genet. 2015 Apr 7;11(4):e1004969. doi: 10.1371/journal.pgen.1004969 (PMC4388571; doi:10.1371/journal.pgen.1004969)
Supplement: S1 Fig — (PDF) [file pgen.1004969.s003.pdf]

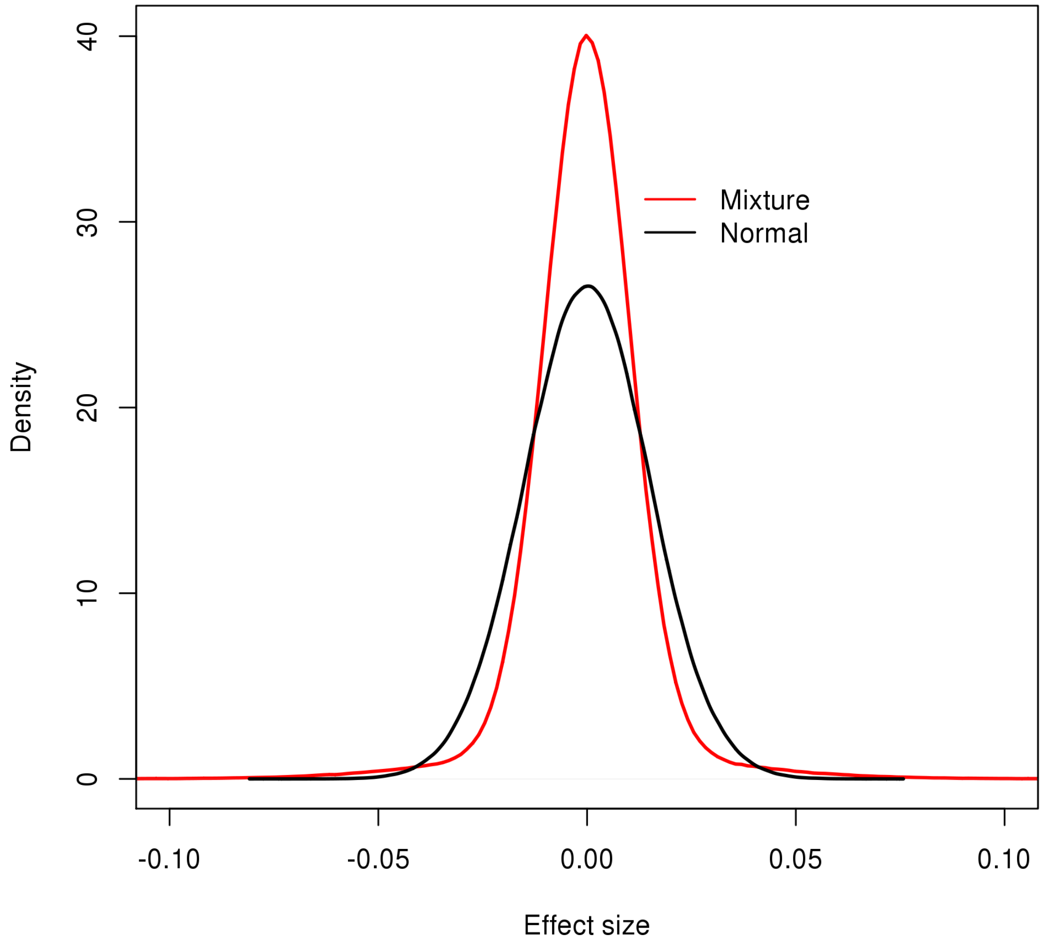

Figure S1. Probability density function of the mixture distribution (red) used to sample SNP effects and probability density function of a normal distribution with the same mean and variance (black).
