# Supplementary material for: Simultaneous Discovery, Estimation and Prediction Analysis of Complex Traits Using a Bayesian Mixture Model
Source: PLoS Genet. 2015 Apr 7;11(4):e1004969. doi: 10.1371/journal.pgen.1004969 (PMC4388571; doi:10.1371/journal.pgen.1004969)
Supplement: S2 Fig — SNPs are ranked according to their contribution to heritability calculated as 2p(1 – p)β 2, where p is the allele frequency and β the effect of the SNP. The total number of causative SNPs was 3, 000 with 10, 310 and 2,680 SNP effects sampled from a zero mean normal distribution with variance 10−2, 10−3, and 10−4, respectively. Trait heritabilities (h 2) were 0.2, 0.5 and 0.8. (PDF) [file pgen.1004969.s004.pdf]

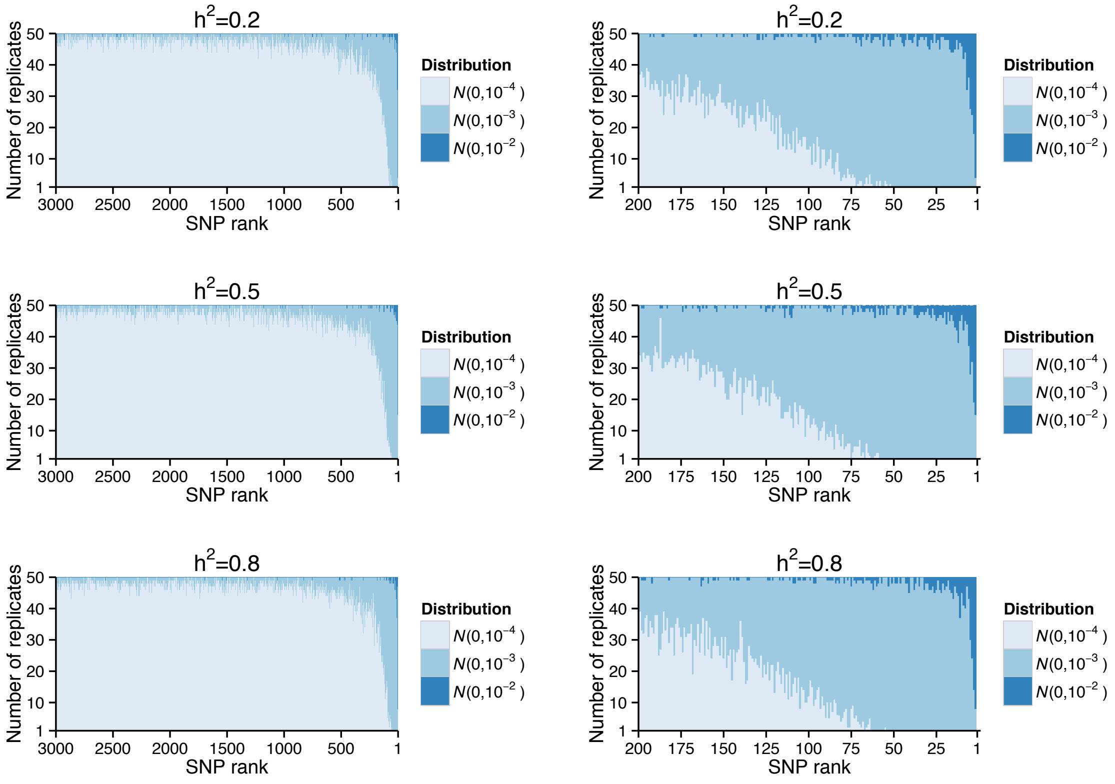

Figure S2. Contribution of causal SNPs to heritability in the simulation scenario using real genotypes. SNPs are ranked according to their contribution to heritability calculated as $2p(1\text{-}p)\beta^2$, where p is the allele frequency and β the effect of the SNP. The plots on the right hand side show a zoomed-in view for the 200 highest ranked SNPs. The total number of causative SNPs was 3, 000 with 10, 310 and 2,680 SNP effects sampled from a zero mean normal distribution with variance 10^{-2}, 10^{-3}, and 10^{-4}, respectively. Trait heritabilities (h^2) were 0.2, 0.5 and 0.8.
