# Supplementary material for: Simultaneous Discovery, Estimation and Prediction Analysis of Complex Traits Using a Bayesian Mixture Model
Source: PLoS Genet. 2015 Apr 7;11(4):e1004969. doi: 10.1371/journal.pgen.1004969 (PMC4388571; doi:10.1371/journal.pgen.1004969)
Supplement: S3 Fig — Shown is the true positive rate as a function of false positive rate for correct identification of 100kb (A) and 500kb (B) regions containing causative SNPs. Simulations are based on real SNP data of 3,924 individuals genotyped for 287,854 SNPs. The total number of causative SNPs was 3,000 with 10 (solid line), 310 (dotted line) and 2,680 effects sampled from a zero mean normal distribution with variance 10−2, 10−3, and 10−4, respectively. Trait heritabilities (h 2) were 0.2, 0.5 and 0.8. (PDF) [file pgen.1004969.s005.pdf]

A. 100kb window size

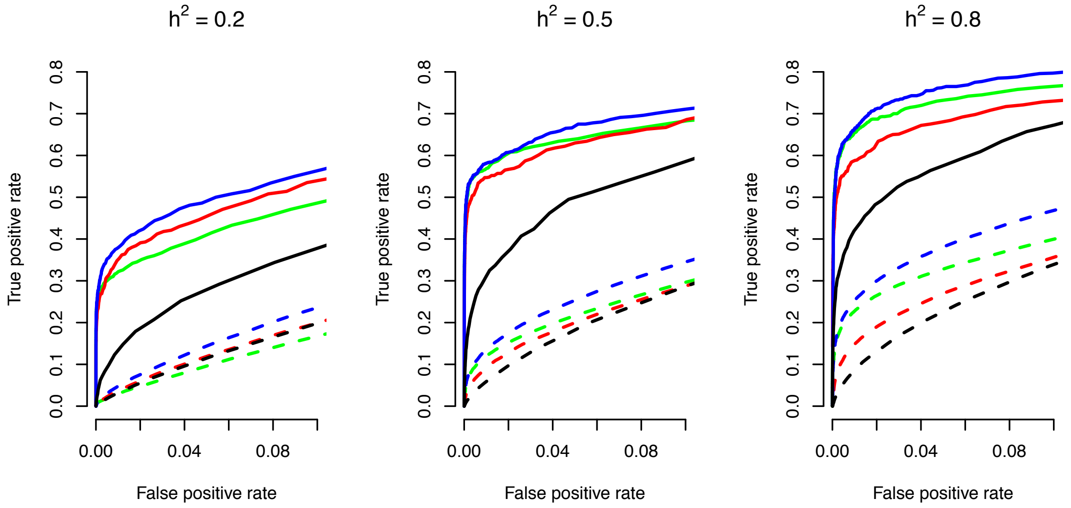

B. 500kb window size

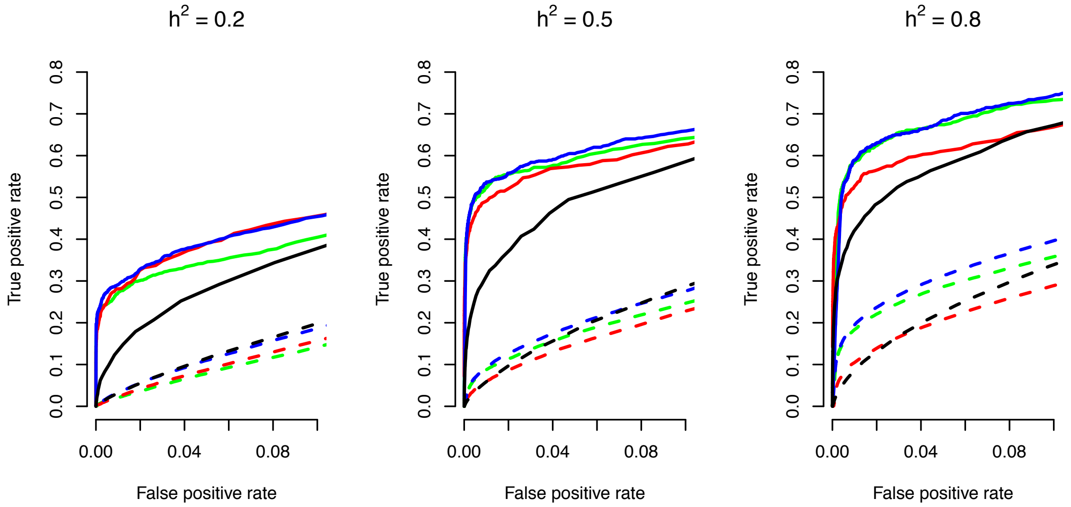

Figure S3. Comparison of causal variant identification accuracy of BayesR, BSLM, LMM and single-SNP analysis in simulated data using 100kb and 500kb regions. Shown is the true positive rate as a function of false positive rate for correct identification of 100kb (A) and 500kb (B) regions containing causative SNPs. Simulations are based on real SNP data of 3,924 individuals genotyped for 287,854 SNPs. The total number of causative SNPs was 3,000 with 10 (solid line), 310 (dotted line) and 2,680 effects sampled from a zero mean normal distribution with variance 10^{-2}, 10^{-3}, and 10^{-4}, respectively. Trait heritabilities (h^2) were 0.2, 0.5 and 0.8.
