# Supplementary material for: Simultaneous Discovery, Estimation and Prediction Analysis of Complex Traits Using a Bayesian Mixture Model
Source: PLoS Genet. 2015 Apr 7;11(4):e1004969. doi: 10.1371/journal.pgen.1004969 (PMC4388571; doi:10.1371/journal.pgen.1004969)
Supplement: S4 Fig — The proportion of variance in each mixture component was calculated as the sum of the square of the sampled effect sizes of the SNPs allocated to each component divided by the sum of the total variance explained by SNPs. Genotype data was simulated for 20,000 uncorrelated SNPs and 5,000 individuals. Genetic architectures ranging from very sparse to highly polygenic were generated by sampling 10, 100, 1,000, 10,000, or 20,0000 SNP effects either from a standard normal distribution or a gamma distribution with shape 0.44 and scale 1.66. Trait heritability was 0.5. Results are based on 50 replicates for each scenario. (PDF) [file pgen.1004969.s006.pdf]

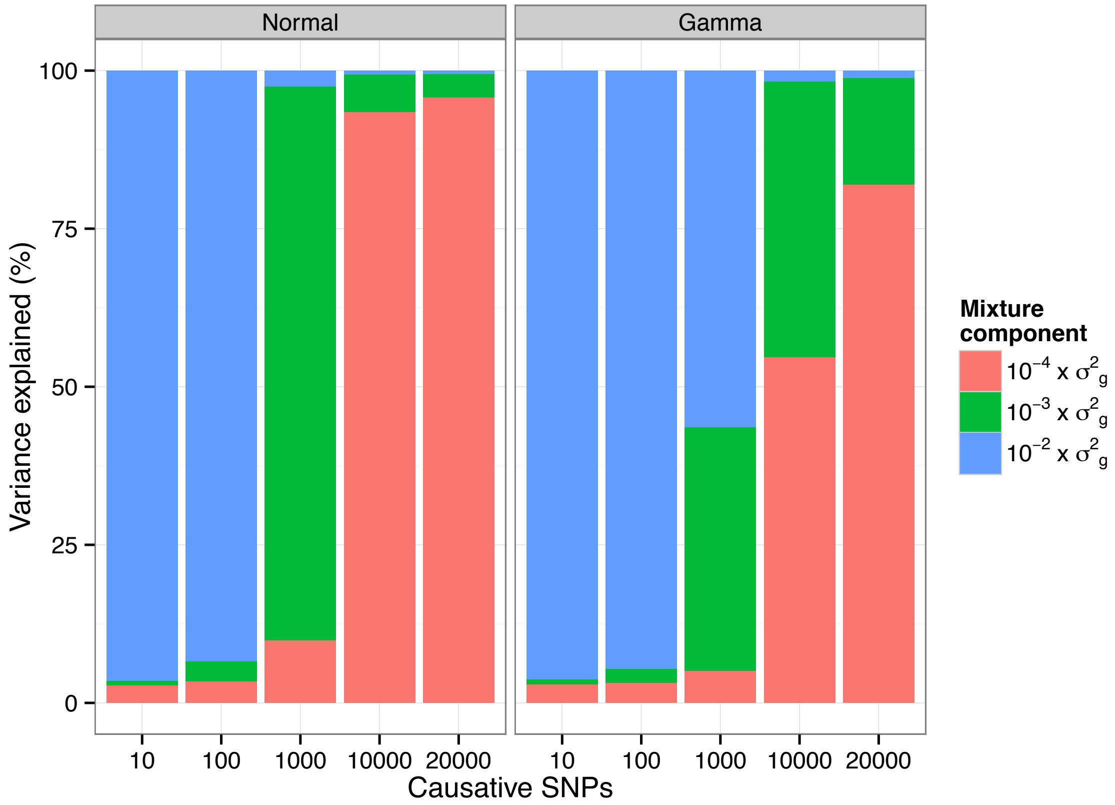

Figure S4. Proportion of total genetic variance explained by each mixture component for different simulated genetic architectures. Genotype data was simulated for 20,000 uncorrelated SNPs and 5,000 individuals. Genetic architectures ranging from very sparse to highly polygenic were generated by sampling 10, 100, 1,000, 10,000, or 20,0000 SNP effects either from a standard normal distribution or a gamma distribution with shape 0.44 and scale 1.66. Trait heritability was 0.5. Results are based on 50 replicates for each scenario.
