# Supplementary material for: Simultaneous Discovery, Estimation and Prediction Analysis of Complex Traits Using a Bayesian Mixture Model
Source: PLoS Genet. 2015 Apr 7;11(4):e1004969. doi: 10.1371/journal.pgen.1004969 (PMC4388571; doi:10.1371/journal.pgen.1004969)
Supplement: S5 Fig — SNPs are ranked according to their contribution to heritability. Genotype data was simulated for 20,000 uncorrelated SNPs and 5,000 individuals. Genetic architectures ranging from very sparse to highly polygenic were generated by sampling 10, 100, 1,000, 10,000, or 20,0000 SNP effects either from (A) a standard normal distribution or (B) a gamma distribution with shape 0.44 and scale 1.66. Trait heritability was 0.5. Results are based on 50 replicates for each case. (PDF) [file pgen.1004969.s007.pdf]

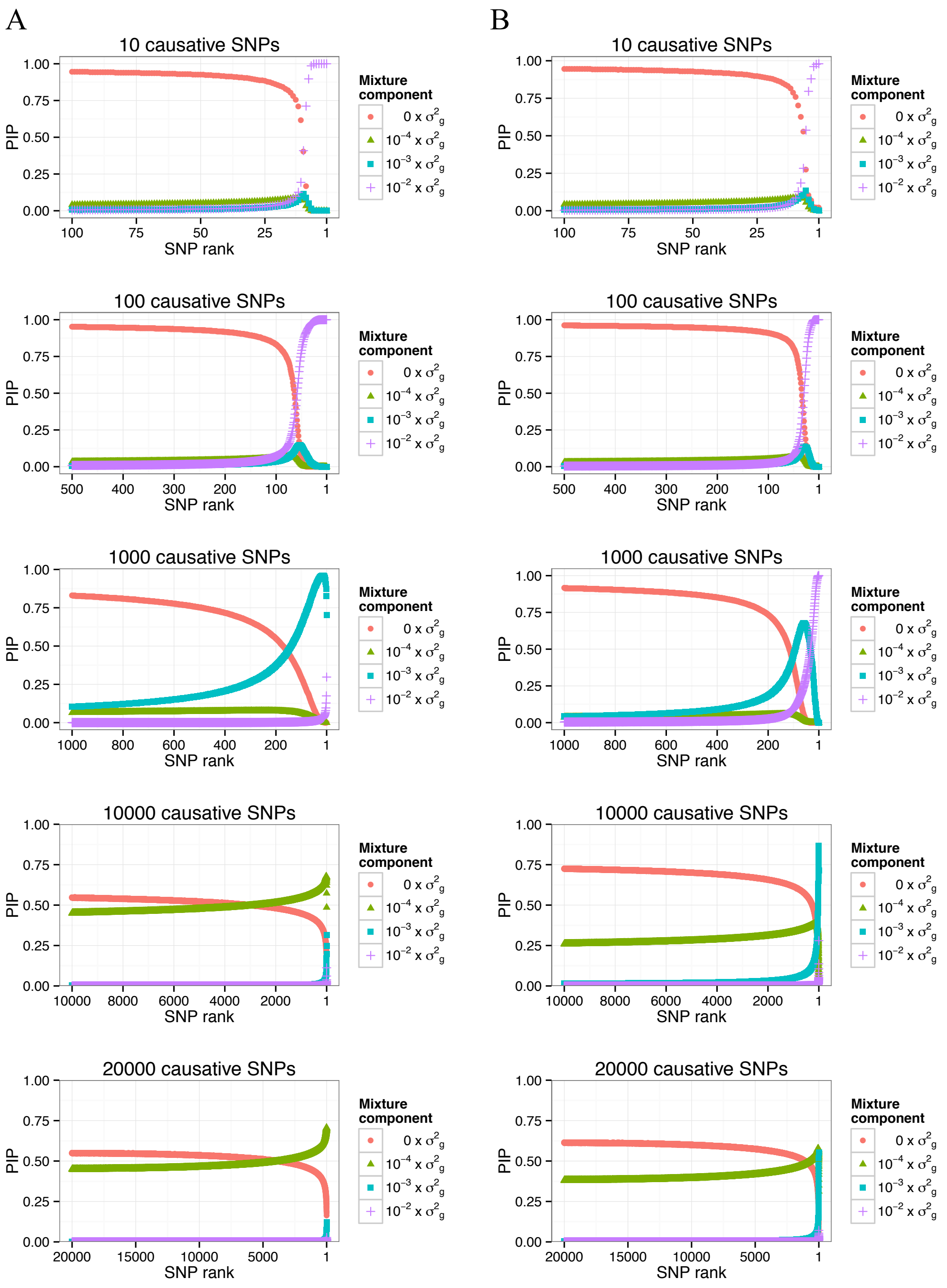

Figure S5. Posterior inclusion probabilities (PIP) of SNPs for different simulated genetic architectures. SNPs are ranked according to their contribution to heritability. Genotype data was simulated for 20,000 uncorrelated SNPs and 5,000 individuals. Genetic architectures ranging from very sparse to highly polygenic were generated by sampling 10, 100, 1,000, 10,000, or 20,0000 SNP effects either from (A) a standard normal distribution or (B) a gamma distribution with shape 0.44 and scale 1.66. Trait heritability was 0.5. Results are based on 50 replicates for each case.
