# Supplementary material for: Simultaneous Discovery, Estimation and Prediction Analysis of Complex Traits Using a Bayesian Mixture Model
Source: PLoS Genet. 2015 Apr 7;11(4):e1004969. doi: 10.1371/journal.pgen.1004969 (PMC4388571; doi:10.1371/journal.pgen.1004969)
Supplement: S6 Fig — Shown is the true positive rate as a function of false positive rate to identify causative SNPs. Genotype data was simulated for 20,000 uncorrelated SNPs and 5,000 individuals. 10, 100, 1,000, or 10,000 SNP effects were sampled either from a standard normal distribution (solid line) or a gamma distribution with shape 0.44 and scale 1.66 (dashed line). Trait heritability was 0.5. Results are based on 50 replicates for each scenario. (PDF) [file pgen.1004969.s008.pdf]

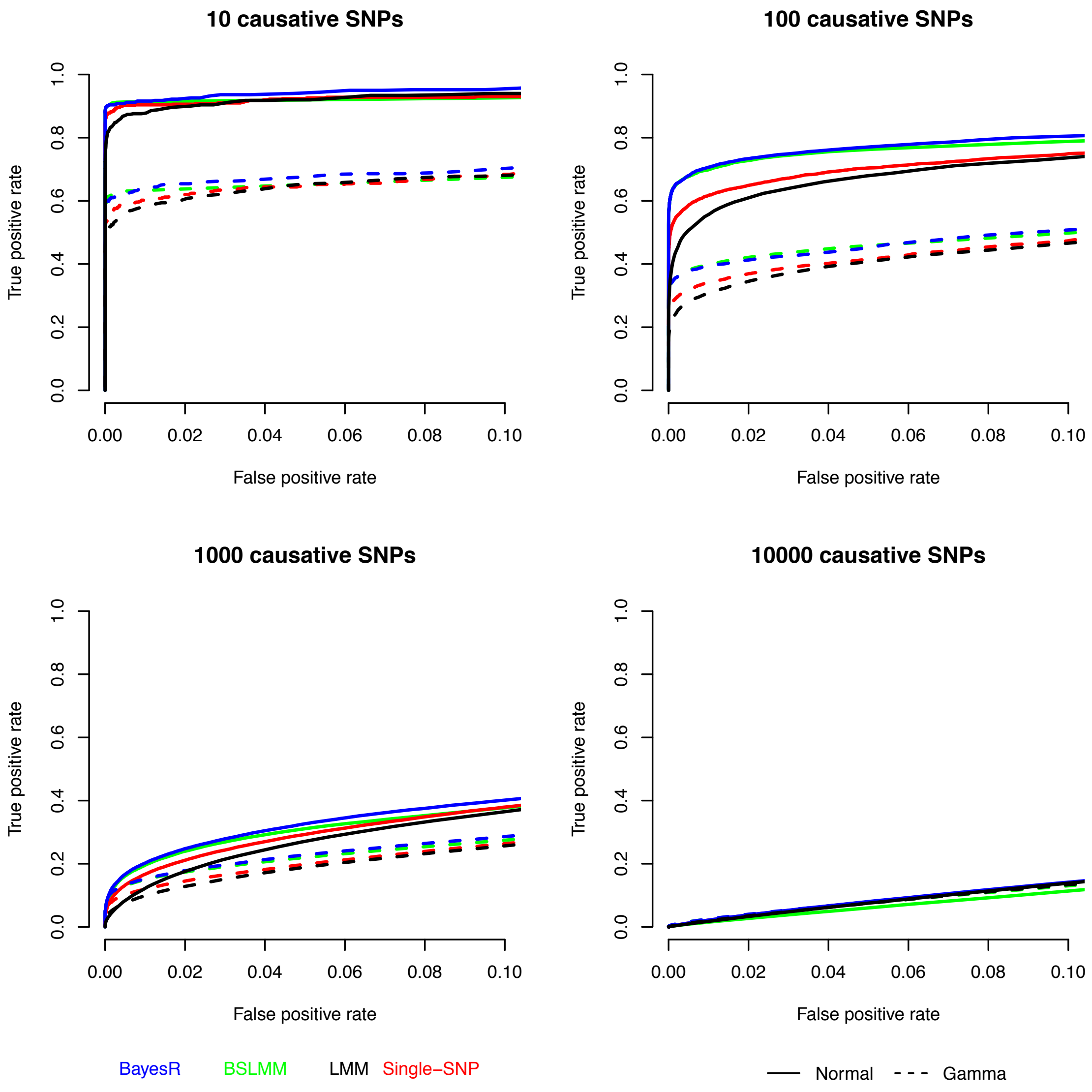

Figure S6. Comparison of causal variant identification accuracy of BayesR, BSLM, LMM and single-SNP analysis for different simulated genetic architectures. Shown is the true positive rate as a function of false positive rate to identify causative SNPs. Genotype data was simulated for 20,000 uncorrelated SNPs and 5,000 individuals. 10, 100, 1,000, or 10,000 SNP effects were sampled either from a standard normal distribution (solid line) or a gamma distribution with shape 0.44 and scale 1.66 (dashed line). Trait heritability was 0.5. Results are based on 50 replicates for each scenario.
