# Supplementary material for: Simultaneous Discovery, Estimation and Prediction Analysis of Complex Traits Using a Bayesian Mixture Model
Source: PLoS Genet. 2015 Apr 7;11(4):e1004969. doi: 10.1371/journal.pgen.1004969 (PMC4388571; doi:10.1371/journal.pgen.1004969)
Supplement: S7 Fig — The variance in each mixture component was calculated as the sum of the square of the sampled effect sizes of SNPs allocated to each component. Vertical lines denote 95% credible intervals. (PDF) [file pgen.1004969.s009.pdf]

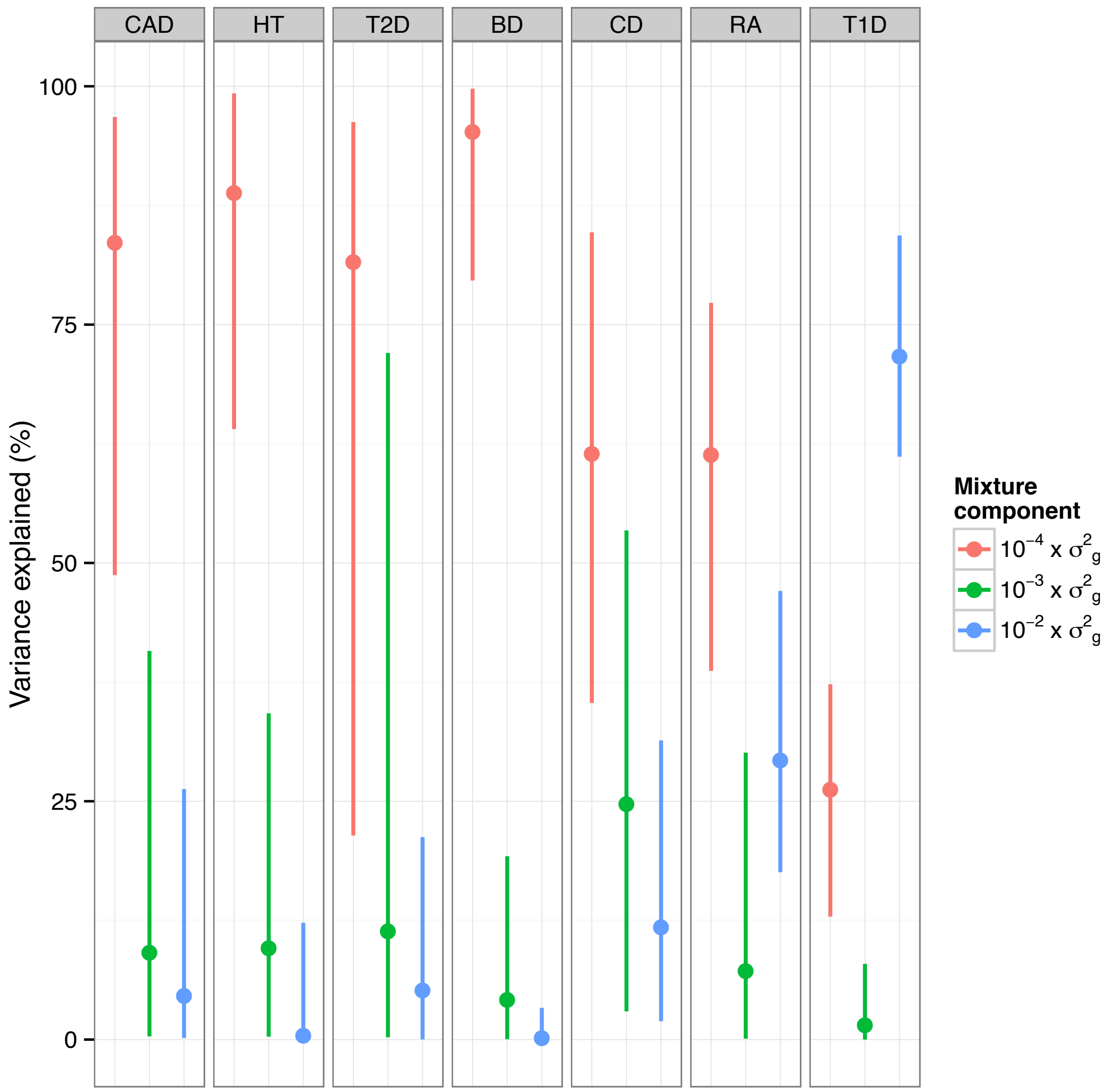

Figure S7. Proportion of the SNP-based variance explained by each mixture component of BayesR for seven traits in WTCCC. The variance in each mixture component was calculated as the sum of the square of the sampled effect sizes of SNPs allocated to each component. Vertical lines denote 95% credible intervals.
