# Supplementary material for: Simultaneous Discovery, Estimation and Prediction Analysis of Complex Traits Using a Bayesian Mixture Model
Source: PLoS Genet. 2015 Apr 7;11(4):e1004969. doi: 10.1371/journal.pgen.1004969 (PMC4388571; doi:10.1371/journal.pgen.1004969)
Supplement: S8 Fig — The MCMC chain was run for 50,000 cycles with the first 20,000 samples discarded as burn-in. Posterior estimates of parameters are based on 3,000 samples drawing every 10th sample after burn-in. None of the four runs indicated apparent convergence problems. (PDF) [file pgen.1004969.s010.pdf]

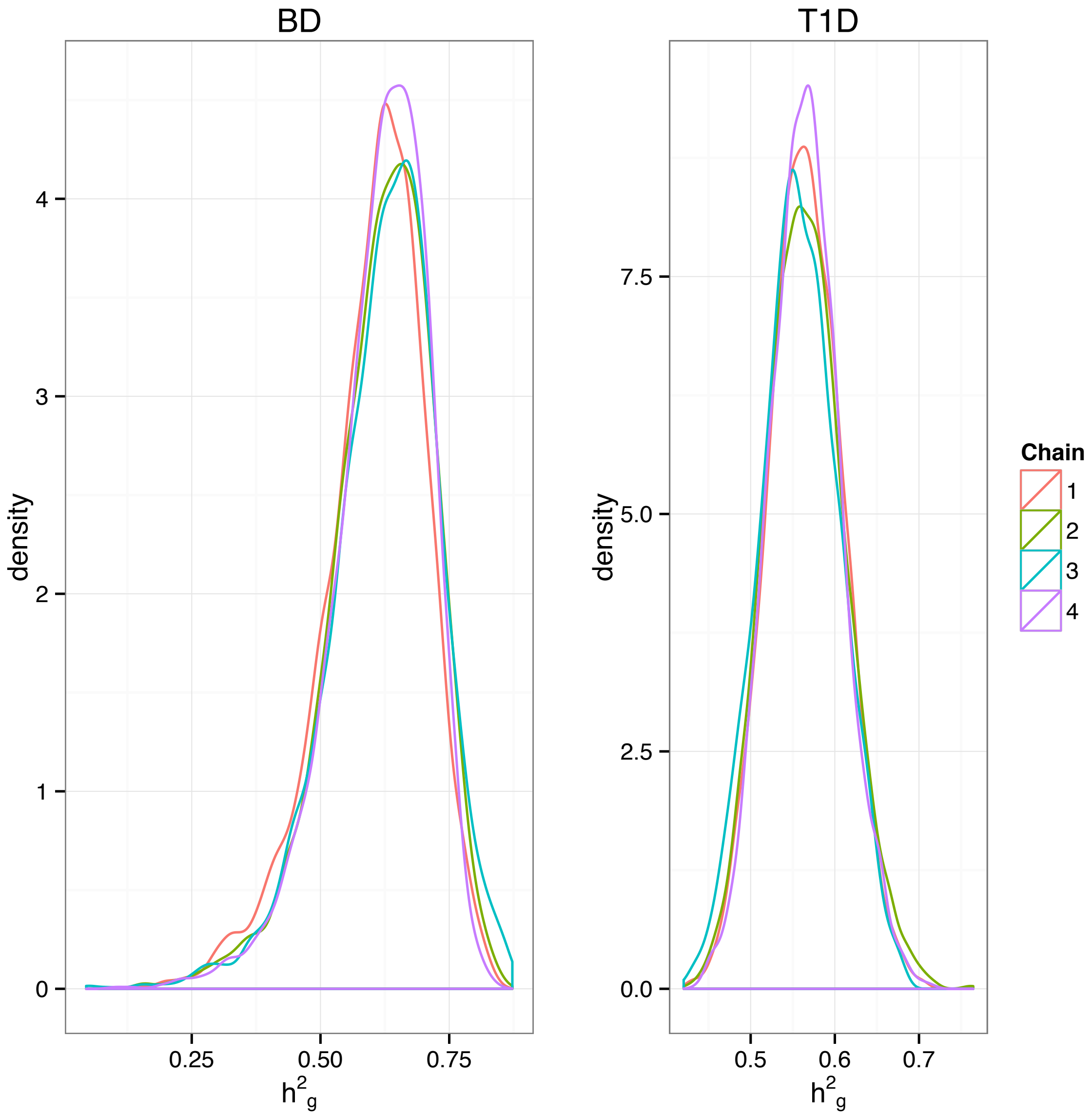

Figure S8. Comparison of posterior density of the SNP-based heritability (h_g^2) of four independent MCMC chains for BD and T1D in WTCCC. The MCMC chain was run for 50,000 cycles with the first 20,000 samples discarded as burn-in. Posterior estimates of parameters are based on 3,000 samples drawing every 10th sample after burn-in. None of the four runs indicated apparent convergence problems.
