# Supplementary material for: Simultaneous Discovery, Estimation and Prediction Analysis of Complex Traits Using a Bayesian Mixture Model
Source: PLoS Genet. 2015 Apr 7;11(4):e1004969. doi: 10.1371/journal.pgen.1004969 (PMC4388571; doi:10.1371/journal.pgen.1004969)
Supplement: S9 Fig — The MCMC chain was run for 50,000 cycles with the first 20,000 samples discarded as burn-in. Posterior estimates of parameters are based on 3,000 samples drawing every 10th sample after burn-in. None of the four runs indicated apparent mixing problems. (PDF) [file pgen.1004969.s011.pdf]

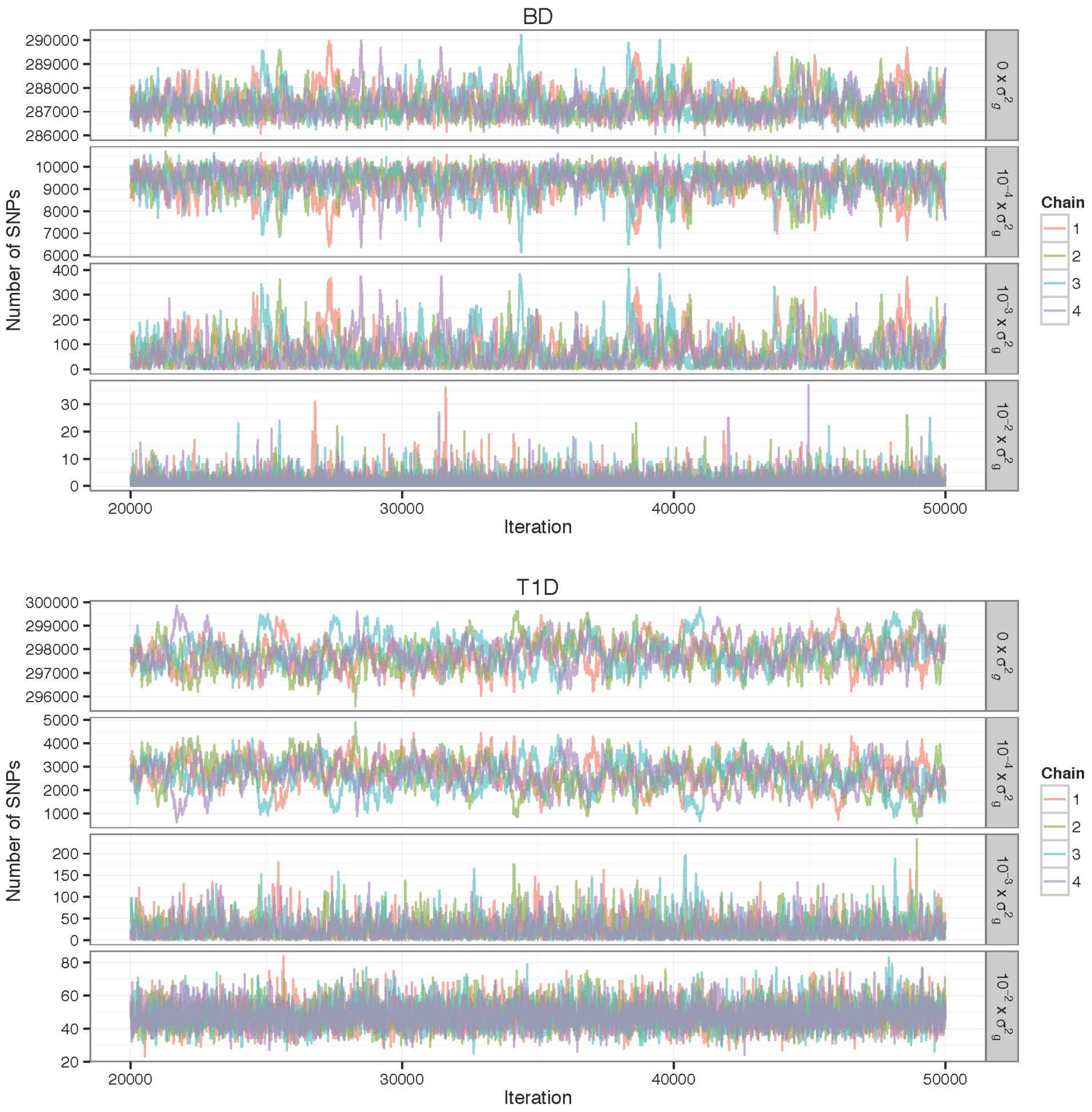

Figure S9. Trace-plots of the posterior number of SNP in each mixture component of four independent MCMC chains for BD and T1D in WTCCC. The MCMC chain was run for 50,000 cycles with the first 20,000 samples discarded as burn-in. Posterior estimates of parameters are based on 3,000 samples drawing every 10th sample after burn-in. None of the four runs indicated apparent mixing problems.
