# Supplementary material for: Simultaneous Discovery, Estimation and Prediction Analysis of Complex Traits Using a Bayesian Mixture Model
Source: PLoS Genet. 2015 Apr 7;11(4):e1004969. doi: 10.1371/journal.pgen.1004969 (PMC4388571; doi:10.1371/journal.pgen.1004969)
Supplement: S1 Table — (PDF) [file pgen.1004969.s012.pdf]

Table S1 Comparison of estimated heritability from BayesR, BSLMM and LMM for different simulated genetic architectures.

	Normal			Gamma		
Causal SNPs	BayesR	BSLMM	LMM	BayesR	BSLMM	LMM
10	0.52 (0.011)	0.52 (0.014)	0.50 (0.047)	0.51 (0.010)	0.52 (0.011)	0.50 (0.041)
100	0.53 (0.012)	0.52 (0.014)	0.51 (0.045)	0.52 (0.014)	0.51 (0.014)	0.50 (0.034)
1000	0.51 (0.023)	0.50 (0.032)	0.50 (0.037)	0.52 (0.018)	0.50 (0.024)	0.50 (0.038)
10000	0.48 (0.043)	0.50 (0.039)	0.50 (0.039)	0.50 (0.040)	0.50 (0.039)	0.50 (0.040)
20000	0.48 (0.040)	0.50 (0.035)	0.50 (0.035)	0.49 (0.043)	0.50 (0.041)	0.50 (0.041)

Genotype data was simulated for 20,000 uncorrelated SNPs and 5,000 individuals. Phenotypes were generated by sampling 10, 100, 1,000, 10,000, or 20,000 SNP effects from a standard normal distribution (Normal) or a gamma distribution (Gamma) with shape 0.44 and scale 1.66. Trait heritability was 0.5. Means and standard deviations (in parenthesis) are based on 50 replicates for each scenario.
