# Supplementary material for: Simultaneous Discovery, Estimation and Prediction Analysis of Complex Traits Using a Bayesian Mixture Model
Source: PLoS Genet. 2015 Apr 7;11(4):e1004969. doi: 10.1371/journal.pgen.1004969 (PMC4388571; doi:10.1371/journal.pgen.1004969)
Supplement: S2 Table — (PDF) [file pgen.1004969.s013.pdf]

Table S2 Comparison of prediction accuracy of BayesR, BSLMM, LMM and GPRS for different simulated genetic architectures.

	Normal				Gamma			
Causal SNPs	BayesR	BSLMM	LMM	GPRS	BayesR	BSLMM	LMM	GPRS
10	0.70 (0.016)	0.71 0.016)	0.22 (0.033)	0.70 (0.017)	0.70 (0.017)	0.70 (0.016)	0.22 (0.032)	0.70 (0.016)
100	0.68 (0.021)	0.68 (0.021)	0.23 (0.033)	0.64 (0.024)	0.69 (0.017)	0.69 (0.016)	0.22 (0.026)	0.67 (0.019)
1000	0.39 (0.028)	0.37 (0.03)	0.21 (0.03)	0.29 (0.034)	0.57 (0.022)	0.57 (0.022)	0.22 (0.032)	0.50 (0.026)
10000	0.22 (0.026)	0.22 (0.026)	0.22 (0.025)	0.21 (0.030)	0.27 (0.029)	0.26 (0.03)	0.22 (0.032)	0.21 (0.034)
20000	0.21 (0.028)	0.22 (0.029)	0.22 (0.029)	0.21 (0.030)	0.23 (0.035)	0.22 (0.034)	0.21 (0.033)	0.20 (0.036)

Prediction accuracy was measured as the correlation between true and predicted phenotypes. Genotype data was simulated for 20,000 uncorrelated SNPs and 5,000 individuals. Phenotypes were generated by sampling 10, 100, 1,000, 10,000, or 20,000 SNP effects from a standard normal distribution (Normal) or a gamma distribution (Gamma) with shape 0.44 and scale 1.66. Trait heritability was 0.5. Means and standard deviations (in parenthesis) are based on 50 replicates for each scenario. In each replicate, the data set was randomly split into a training sample containing 80% of individuals and a validation sample containing the remaining 20%.
