# Supplementary material for: Simultaneous Discovery, Estimation and Prediction Analysis of Complex Traits Using a Bayesian Mixture Model
Source: PLoS Genet. 2015 Apr 7;11(4):e1004969. doi: 10.1371/journal.pgen.1004969 (PMC4388571; doi:10.1371/journal.pgen.1004969)
Supplement: S3 Table — (PDF) [file pgen.1004969.s014.pdf]

Table S3 Comparison of SNP-based heritability estimates of BayesR, BSLMM and LMM in WTCCC data on the liability scale.

Disease	Prevalence (%)	BayesR	BSLMM	LMM
BD [1]	0.45	0.30 (0.049)	0.31 (0.029)	0.31 (0.029)
CAD [2]	5.6	0.33 (0.119)	0.37 (0.055)	0.39 (0.055)
CD [3]	0.1	0.18 (0.030)	0.18 (0.023)	0.20(0.023)
HT [4]	26.4	0.60 (0.177)	0.65 (0.089)	0.66 (0.089)
RA [5]	1	0.23 (0.047)	0.24 (0.029)	0.29 (0.035)
T1D [6]	0.54	0.28 (0.020)	0.27 (0.020)	0.32 (0.030)
T2D [7]	3	0.31 (0.092)	0.33 (0.046)	0.34 (0.046)

Values in parentheses are standard errors. The number of cases in the WTCCC case-control sample is much larger than the prevalence in the population. We accounted for ascertainment in the sample and transferred the heritability estimates on the observed scale on a scale of liability using the approach of Lee et al. [8].

1. Lichtenstein P, Yip BH, Bjork C, Pawitan Y, Cannon TD, et al. (2009) Common genetic determinants of schizophrenia and bipolar disorder in Swedish families: a population-based study. Lancet 373: 234-239.
2. Marenberg ME, Risch N, Berkman LF, Floderus B, de Faire U (1994) Genetic susceptibility to death from coronary heart disease in a study of twins. N Engl J Med 330: 1041-1046.
3. The Welcome Trust Case Control Consortium (2007) Genome-wide association study of 14,000 cases of seven common diseases and 3,000 shared controls. Nature 447: 661-678.
4. Kearney PM, Whelton M, Reynolds K, Muntner P, Whelton PK, et al. (2005) Global burden of hypertension: analysis of worldwide data. The Lancet 365: 217-223.
5. Symmons D, Turner G, Webb R, Asten P, Barrett E, et al. (2002) The prevalence of rheumatoid arthritis in the United Kingdom: new estimates for a new century. Rheumatology (Oxford) 41: 793-800.
6. Hyttinen V, Kaprio J, Kinnunen L, Koskenvuo M, Tuomilehto J (2003) Genetic liability of type 1 diabetes and the onset age among 22,650 young Finnish twin pairs: a nationwide follow-up study. Diabetes 52: 1052-1055.
7. Das SK, Elbein SC (2006) The Genetic Basis of Type 2 Diabetes. Cellscience 2: 100-131.
8. Lee SH, Wray NR, Goddard ME, Visscher PM (2011) Estimating missing heritability for disease from genome-wide association studies. Am J Hum Genet 88: 294-305.
