# Supplementary material for: Simultaneous Discovery, Estimation and Prediction Analysis of Complex Traits Using a Bayesian Mixture Model
Source: PLoS Genet. 2015 Apr 7;11(4):e1004969. doi: 10.1371/journal.pgen.1004969 (PMC4388571; doi:10.1371/journal.pgen.1004969)
Supplement: S4 Table — (PDF) [file pgen.1004969.s015.pdf]

Table S4 Percentage of total variance on the liability scale explained in WTCCC data, corrected for ascertainment.

Disease	Prevalence (%)	BayesR	BSLMM	LMM	GRPS
BD [1]	0.45	2.1 (0.32)	2.1 (0.33)	2.1 (0.33)	2.2 (0.48)
CAD [2]	5.6	1.8 (0.24)	2.1 (0.22)	1.4 (0.19)	1.1 (0.54)
CD [3]	0.1	2.5 (0.31)	2.8 (0.30)	1.1 (0.41)	1.6 (0.58)
HT [4]	26.4	2.8 (0.33)	3.0 (0.33)	3.0 (0.34)	3.0 (0.50)
RA [5]	1	7.5 (0.23)	7.4 (0.27)	1.5 (0.35)	5.8 (0.57)
T1D [6]	0.54	22.9 (0.18)	22.0 (0.16)	2.2 (0.28)	12.9 (0.39)
T2D [7]	3	1.7 (0.33)	1.8 (0.26)	1.3 (0.28)	1.1 (0.66)

Values in parentheses are standard errors. Computations are based on *formula 15* in Lee *et al.*[8]. R^2 on the observed scale was computed as the squared correlation of predicted and true phenotype of the prediction analyses.

1. Lichtenstein P, Yip BH, Bjork C, Pawitan Y, Cannon TD, et al. (2009) Common genetic determinants of schizophrenia and bipolar disorder in Swedish families: a population-based study. Lancet 373: 234-239.
2. Marenberg ME, Risch N, Berkman LF, Floderus B, de Faire U (1994) Genetic susceptibility to death from coronary heart disease in a study of twins. N Engl J Med 330: 1041-1046.
3. The Welcome Trust Case Control Consortium (2007) Genome-wide association study of 14,000 cases of seven common diseases and 3,000 shared controls. Nature 447: 661-678.
4. Kearney PM, Whelton M, Reynolds K, Muntner P, Whelton PK, et al. (2005) Global burden of hypertension: analysis of worldwide data. The Lancet 365: 217-223.
5. Symmons D, Turner G, Webb R, Asten P, Barrett E, et al. (2002) The prevalence of rheumatoid arthritis in the United Kingdom: new estimates for a new century. Rheumatology (Oxford) 41: 793-800.
6. Hyttinen V, Kaprio J, Kinnunen L, Koskenvuo M, Tuomilehto J (2003) Genetic liability of type 1 diabetes and the onset age among 22,650 young Finnish twin pairs: a nationwide follow-up study. Diabetes 52: 1052-1055.
7. Das SK, Elbein SC (2006) The Genetic Basis of Type 2 Diabetes. Cellscience 2: 100-131.
8. Lee SH, Goddard ME, Wray NR, Visscher PM (2012) A better coefficient of determination for genetic profile analysis. Genet Epidemiol 36: 214-224.
