# Supplementary material for: Simultaneous Discovery, Estimation and Prediction Analysis of Complex Traits Using a Bayesian Mixture Model
Source: PLoS Genet. 2015 Apr 7;11(4):e1004969. doi: 10.1371/journal.pgen.1004969 (PMC4388571; doi:10.1371/journal.pgen.1004969)
Supplement: S5 Table — (PDF) [file pgen.1004969.s016.pdf]

Table S5 Estimates of model size and number of SNPs in each mixture component by BayesR for seven traits in WTCCC.

Trait	Model size		Number of SNPs in mixture component							
			$0 \times \sigma^2_g$		$10^{-4} \times \sigma^2_g$		$10^{-3} \times \sigma^2_g$		$10^{-2} \times \sigma^2_g$	
CAD	8141	(1436)	292782	(1436)	7995	(1563)	138	(144)	8	(8.9)
HT	8472	(1074)	290192	(1074)	8314	(1179)	154	(119)	4	(6.8)
T2D	8198	(1236)	290745	(1236)	8057	(1353)	133	(131)	9	(7.5)
BD	9411	(540)	287307	(540)	9345	(592)	65	(60)	2	(2.7)
CD	6265	(1287)	299702	(1287)	5967	(1421)	281	(153)	17	(9.8)
RA	5059	(1608)	295134	(1608)	4844	(1746)	185	(161)	29	(11.1)
T1D	2633	(575)	297916	(575)	2560	(584)	26	(25)	48	(7.6)

Values in parenthesis are standard deviations of posterior samples.
