# Supplementary material for: Simultaneous Discovery, Estimation and Prediction Analysis of Complex Traits Using a Bayesian Mixture Model
Source: PLoS Genet. 2015 Apr 7;11(4):e1004969. doi: 10.1371/journal.pgen.1004969 (PMC4388571; doi:10.1371/journal.pgen.1004969)
Supplement: S6 Table — (PDF) [file pgen.1004969.s017.pdf]

Table S6 Posterior means from BayesR using the full (All SNPs) and a reduced MCMC scheme (500 SNPs).

Trait	Parameter		Posterior Mean			
			All SNPs		500 SNPs	
BD	h_g^2		0.60	(0.048)	0.56	(0.054)
	AUC		0.61	(0.014)	0.61	(0.014)
	Model size		8891	(574)	8730	(770)
	Number of SNPs	$0 \times \sigma_g^2$	287827	(574)	287988	(770)
		$10^{-4} \times \sigma_g^2$	8774	(636)	8602	(854)
		$10^{-3} \times \sigma_g^2$	114	(60.8)	123	(83.8)
		$10^{-2} \times \sigma_g^2$	3	(0.6)	4.3	(1.0)
T1D	h_g^2		0.56	(0.025)	0.56	(0.029)
	AUC		0.85	(0.011)	0.85	(0.011)
	Model size		2363	(299)	2410	(367)
	Number of SNPs	$0 \times \sigma_g^2$	298186	(299)	298140	(367)
		$10^{-4} \times \sigma_g^2$	2268	(310)	2315	(377)
		$10^{-3} \times \sigma_g^2$	46	(12.9)	46	(12.6)
		$10^{-2} \times \sigma_g^2$	48	(1.5)	49	(1.7)

Results are means and (standard deviation) based on the analyses of 10 replicates used in the prediction analysis. Sampling scheme 'All SNPs' updates every SNP effect in each MCMC iteration. Scheme '500 SNPs' uses a modified sampling scheme. The results showed only minor differences in the posterior parameter estimates between the two MCMC sampling schemes.
